# The *Pseudomonas syringae* type III effector HopG1 triggers necrotic cell death that is attenuated by AtNHR2B

**DOI:** 10.1038/s41598-022-09335-1

**Published:** 2022-03-30

**Authors:** Catalina Rodríguez-Puerto, Rupak Chakraborty, Raksha Singh, Perla Rocha-Loyola, Clemencia M. Rojas

**Affiliations:** 1 Department of Entomology and Plant Pathology, University of Arkansas, Fayetteville, AR 72703 USA; 2 Present Address: Crop Production and Pest Control Research Unit, U.S. Department of Agriculture-Agricultural Research Service (USDA-ARS), West Lafayette, IN 47907 USA; 3 Present Address: Department of Agronomy, Horticulture and Plant Science, South Dakota State University, Brookings, SD USA

**Keywords:** Microbiology, Plant sciences

## Abstract

The plant pathogenic bacterium *Pseudomonas syringae* pv. tomato DC3000 (*Pst* DC3000) has become a paradigm to investigate plant-bacteria interactions due to its ability to cause disease in the model plant *Arabidopsis thaliana. Pst* DC3000 uses the type III secretion system to deliver type III secreted effectors (T3SEs) directly into the plant cytoplasm. *Pst* DC3000 T3SEs contribute to pathogenicity by suppressing plant defense responses and targeting plant’s physiological processes. Although the complete repertoire of effectors encoded in the *Pst* DC3000 genome have been identified, the specific function for most of them remains to be elucidated. Among those effectors, the mitochondrial-localized T3E HopG1, suppresses plant defense responses and promotes the development of disease symptoms. Here, we show that HopG1 triggers necrotic cell death that enables the growth of adapted and non-adapted pathogens. We further showed that HopG1 interacts with the plant immunity-related protein AtNHR2B and that AtNHR2B attenuates HopG1- virulence functions. These results highlight the importance of HopG1 as a multi-faceted protein and uncover its interplay with AtNHR2B.

## Introduction

*Pseudomonas syringae* is a plant pathogenic Gram-negative bacterium that causes diseases in a wide range of plants. Due to this broad host range, the species has been divided into more than 50 pathovars (pv), each pathovar designation based on their host of isolation^1^. Among those pathovars, *Pseudomonas syringae* pv. tomato DC3000 (*Pst* DC3000), the causal agent of bacterial speck on tomato, has become a model pathogen to understand bacterial pathogenicity towards plants because it can also cause disease in model plants. Wild type *Pst* DC3000 causes disease in the model plant *Arabidopsis thaliana*, and the *Pst* DC3000 *hopQ1-1* mutant is able to cause disease in *Nicotiana benthamiana*^1–4^*.* The pathogenicity of *Pst* DC3000 is mostly due to the type III secretion system (T3SS), a complex of proteins encoded by the Hypersensitive Response and Pathogenicity/ Hypersensitive Response and Conserved (*Hrp/Hrc*) genes^5^. *Hrp/Hrc-* encoded proteins assemble an apparatus spanning the inner and outer bacterial membranes that enables the bacterium to deliver bacterial proteins (effectors), directly into the host cytoplasm^6–8^.

The genome of *Pst* DC3000 encodes 28 type III effectors (T3Es) that are delivered into plant cells^9–11^, where acting together interfere with plant immune responses to facilitate bacterial parasitism^12–14^. The plant immune responses include two main branches: (1) Pathogen-Associated Molecular Patterns-Triggered Immunity (PTI) that recognizes conserved features in pathogens known as Pathogen-Associated Molecular Patterns (PAMPs) through surface-localized Pattern Recognition Receptors (PRRs)^15–17^, and (2) Effector-Triggered Immunity (ETI), that recognizes pathogen effector molecules by R (resistance) proteins^15,16,18^. An outcome of ETI is the elicitation of the hypersensitive response (HR), a type of localized programmed cell death that restricts pathogen proliferation and tissue damage^19–21^.

*Pst* DC3000 T3Es are functionally redundant, as mutations in individual effectors do not have a significant impact on pathogenicity, yet all the effectors are required for full pathogenicity in tomato and *Arabidopsis*^22^. Interestingly, deletion of the T3E HopQ1-1 expands the host range of *Pst* DC3000 to *N. benthamiana*^4^*.* Remarkably, only eight T3Es effectors: AvrPtoB, HopM1, AvrE, HopE1, HopG1, HopAM1-1, HopAA1-1 and HopN1 make up the minimal functional set of effectors needed for *Pst* DC3000 to be virulent in *N. benthamiana*^23^. Among those effectors, HopG1 was previously implicated in the suppression of PTI and ETI responses^24–26^. Further research showed that HopG1 localizes to mitochondria^24^, where it interacts with the mitochondrial-localized kinesin motor protein to modulate actin cytoskeleton and promote the development of chlorotic symptoms^27^.

We previously identified AtNHR2B (*Arabidopsis thaliana* nonhost resistance 2B) as a protein that functions in plant immunity^28–31^. *Atnhr2b* mutant plants are immunocompromised, and as a result, they are susceptible to pathogens that normally do not cause disease in wild-type plants, called non-adapted pathogens^31^. We further showed by live-cell imaging that AtNHR2B tagged with the green fluorescent protein (GFP) localizes to chloroplasts and compartments of the endomembrane system^31^, and through proteomics approaches, we also showed that AtNHR2B interacts with proteins localized to chloroplasts, mitochondria and nucleus^32^.

Here, we show that the *Pst* DC3000 T3E HopG1 interacts with AtNHR2B and that AtNHR2B interferes with HopG1 virulence functions. We also expand the range of functions for HopG1 with the discovery that transient or stable expression of *HopG1* in *N. benthamiana* and Arabidopsis, respectively, triggers necrotic cell death that enables the growth of adapted and non-adapted pathogens. This necrotic cell death is a virulence function that confers advantage to the pathogen, rather than an HR-type of programmed cell death that confers resistance to the plant. These results highlight the importance of HopG1 as a multifaceted protein and uncover the interplay between HopG1 and AtNHR2B.

## Results

### Adapted pathogens changed the pattern of localization and abundance of AtNHR2B-GFP

**Figure 1:**
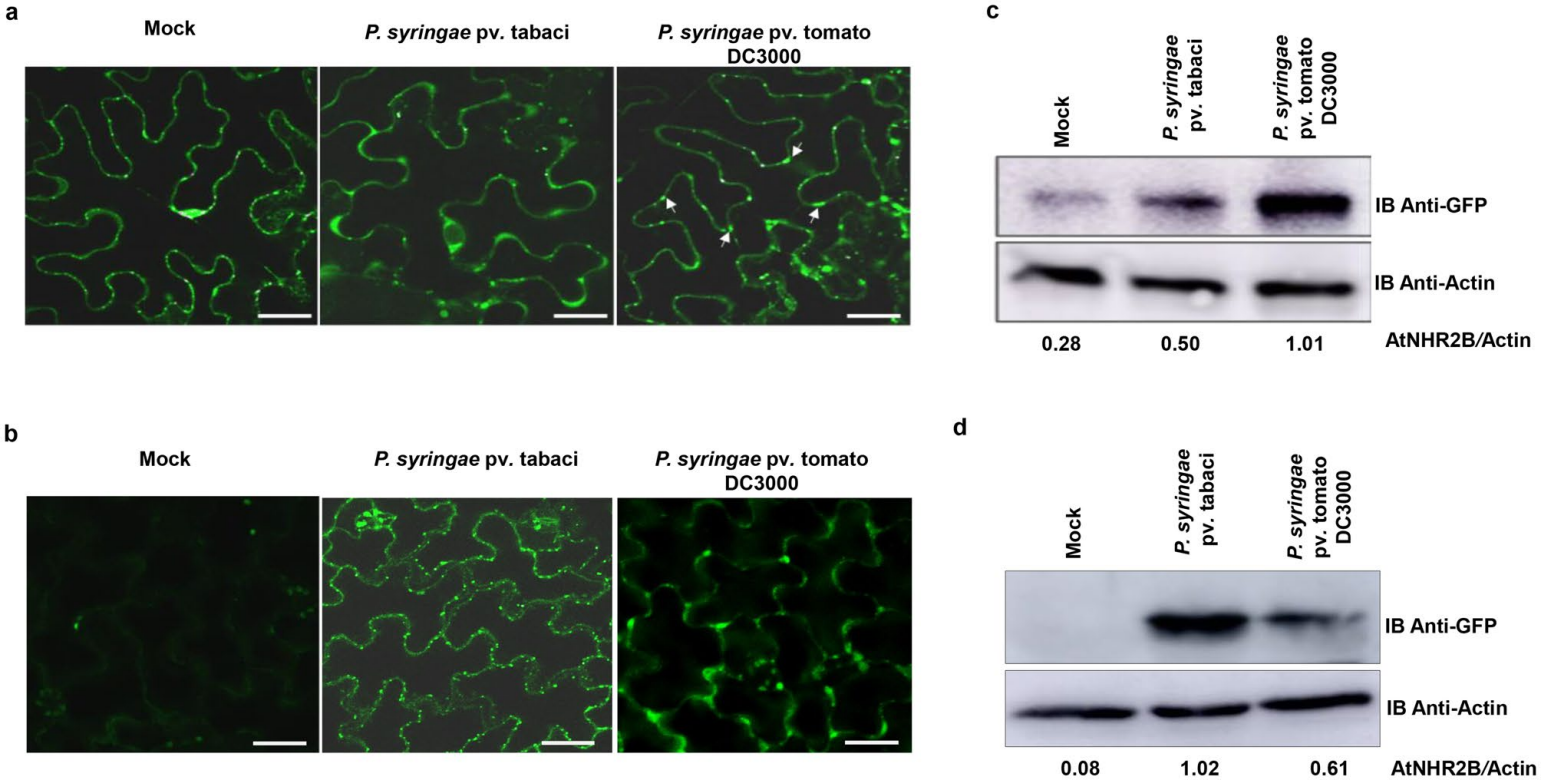
Adapted pathogens interfere with AtNHR2B-GFP localization and protein abundance. (**a**) The host adapted pathogen *Pstab* alters AtNHR2B-GFP localization. *N. benthamiana* plants transiently expressing *AtNHR2B-GFP* were infiltrated with water (mock), or inoculated with either the adapted pathogen *Pstab* or the non-adapted pathogen *Pst* DC3000 at 1 × 10^6^ CFU/ml*.* Treated leaf samples were collected after 24 hpi and imaged by laser scanning confocal microscopy. Confocal images were taken at ×20 magnification and using an excitation and emission wavelengths of 488 nm and 500–535 nm, respectively. White arrows show punctate bodies. Scale bar = 10 µm. (**b**) The host adapted pathogen *Pst* DC3000 alters AtNHR2B-GFP localization in *Arabidopsis. Arabidopsis* transgenic plants expressing *AtNHR2B-GFP* were mock-treated or inoculated with the adapted pathogen *Pst* DC3000 and the non-adapted pathogen *Pstab* at 1 × 10^6^ CFU/ml*.* Treated leaf samples were collected after 24 hpi and imaged by laser scanning confocal microscopy. Confocal images were taken at ×20 magnification and using an excitation and emission wavelengths of 488 nm and 500–535 nm, respectively. White arrows show punctate bodies. Scale bar = 10 µm. (**c**) The host adapted pathogen *Pstab* alters AtNHR2B-GFP protein abundance in tobacco plants. *N. benthamiana* plants transiently expressing *AtNHR2B-GFP* were infiltrated with water (mock) or inoculated with either the adapted pathogen *Pstab* or the non-adapted pathogen *Pst* DC3000 as described for (**a**). Treated leaf samples were collected after 24 hpi to evaluate protein abundance by Western blot using anti-GFP and anti-actin antibodies. Protein quantification was done by ImageJ software (http://rsb.info.nih.gov/ij/), using actin signal for normalization. Number below the protein bands represent the AtNHR2-GFP/Actin ratios. (**d**) The host adapted pathogen *Pst* DC3000 alters AtNHR2B-GFP protein abundance in *Arabidopsis* plants. *A. thaliana* transgenic plants expressing *AtNHR2B-GFP* were infiltrated with water (mock), or inoculated with either the adapted pathogen *Pst* DC3000 or the non-adapted pathogen *Pstab* as described for (**b**)*.* Treated leaf samples were collected after 24 hpi to quantify protein abundance by Western blot using anti-GFP and anti-Actin antibodies. Protein quantification was done as described for (**c**).

We previously showed that *AtNHR2B* is induced by pathogens and pathogen-derived elicitors, and that *AtNHR2B-GFP* transiently expressed in *N. benthamiana* localizes to the cytoplasm and to highly dynamic structures (punctae) reminiscent of subcellular compartments of the endomembrane system^31^. *AtNHR2B* is induced by non-adapted bacterial pathogens (that are unable to cause disease in a particular host), as well as, adapted bacterial pathogens (that cause disease in a specific host); however, we do not know if pathogens’ lifestyle (non-adapted vs adapted) have an effect on the function of AtNHR2B. Since AtNHR2B is an immune-related protein, we hypothesized that adapted pathogens could target this protein for parasitism. To start testing that hypothesis, we evaluated the localization of AtNHR2B-GFP by transient expression in *N. benthamiana*. *N. benthamiana* plants transiently expressing *AtNHR2B-GFP* were mock-treated with water, inoculated with *P. syringae* pv. tabaci (*Pstab*) (adapted pathogen of *N. benthamiana*), or with *Pst* DC3000 (non-adapted pathogen of *N. benthamiana*). Mock-treated plants and plants inoculated with *Pst* DC3000 showed the expected localization to cytoplasm and punctae. Interestingly, after inoculation with *Pstab*, the signal from AtNHR2B-GFP was limited to the cytoplasm (Fig. 1a).

The change in AtNHR2B-GFP pattern of localization in *N. benthamiana* associated with inoculation with the adapted pathogen was validated in transgenic *Arabidopsis* plants expressing *AtNHR2B-GFP* from its native promoter^31^. In such plants, the fluorescent signal was very low in mock-treated plants, but significantly increased upon inoculation with *Pst* DC3000 and *Pstab*. Similar to the results obtained in *N. benthamiana,* the pattern of AtNHR2B-GFP localization differed depending on the pathogen used for the inoculation; inoculation with the non-adapted pathogen of *A. thaliana, Pstab*, caused the localization of AtNHR2B-GFP to cytoplasm and small punctae as previously described^31^ (Fig. 1b). However, inoculation with the adapted pathogen of *A. thaliana*, *Pst* DC3000, changed the pattern of AtNHR2B-GFP localization and the fluorescence signal was detected in the cytoplasm only and appeared diffused and distorted (Fig. 1b).

To evaluate if the diffusion of the fluorescence signal was due to adapted pathogens interfering with protein abundance, we collected leaves from *N. benthamiana* and *Arabidopsis* plants expressing *AtNHR2B-GFP* that were mock-treated, or inoculated with their corresponding adapted and non-adapted pathogens. Collected leaves were used for protein extraction and quantitative Western blot analysis using anti-GFP antibodies to detect AtNHR2B-GFP and anti-actin antibodies for normalization. *N. benthamiana* expressing *AtNHR2B-GFP* and mock-treated, showed low levels of AtNHR2B-GFP. However, inoculation with both adapted (*Pstab*) and non-adapted (*Pst* DC3000) pathogens increased AtNHR2B-GFP concentration in comparison with mock-treated plants, but the amount of AtNHR2B-GFP was reduced by 50% in plant samples treated with *Pstab* in comparison with plants treated with *Pst* DC3000 (Fig. 1c, Supplementary Fig. 1). In a similar way, AtNHR*2B-GFP* transgenic *Arabidopsis* lines did not accumulate any protein after mock treatment and showed ~ 40% less AtNHR2B-GFP after inoculation with the adapted pathogen *Pst* DC3000 than plants inoculated with *Pstab* (Fig. 1d. Supplementary Fig. 2). Altogether, these results revealed that adapted pathogens alter AtNHR2B-GFP protein localization and abundance.

### The *Pst* DC3000 mutant lacking *HopG1* is able to grow better in the *Atnhr2b* mutant background

**Figure 2:**
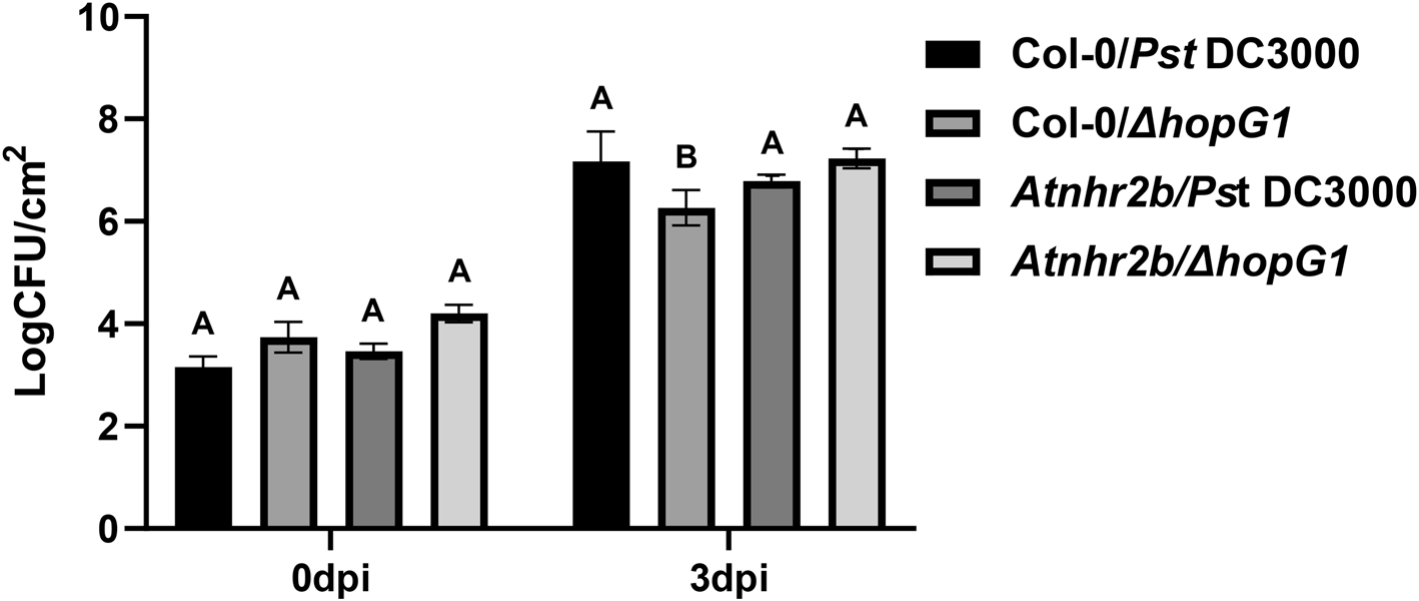
The growth defect in *Pst* DC3000 *hopG1* deletion mutant is restored in *Atnhr2b* mutant *Arabidopsis* plants. Wild-type Col-0 and the *Atnhr2b* mutant were syringe-inoculated with the wild-type *Pst* DC3000 and the *Pst* DC3000 Δ *hopG1* mutant at a concentration of 1 × 10^4^ CFU/ml. Inoculated leaves were collected at 0 and 3 dpi to enumerate bacterial populations. Bars represent means and standard deviation for *Pst* DC3000 and Δ*hopG1* bacterial populations in Col-0 and *Atnhr2b* plants for four replications. Different letters above the bars represent statistically significant difference in bacterial populations of wild-type *Pst* DC3000 and the *Pst* DC3000 Δ *hopG1* in Col-0 versus their respective growth in the *Atnhr2b* mutant plants using a one-way ANOVA and Tukey’s multiple comparison test with P-value ≤ 0.05. The experiments were repeated three times with consistent results.

The finding that the localization and protein abundance of AtNHR2B is altered by adapted pathogens, prompted us to investigate whether AtNHR2B could be a target for *Pst* DC3000 T3E. The finding that HopG1 localizes to mitochondria^24,27^, together with our previous result that AtNHR2B interacts with proteins localized to mitochondria^32^, led us to hypothesize that HopG1 could target AtNHR2B. To test that hypothesis, we initially evaluated the growth of wild-type *Pst* DC3000 and the *Pst* DC3000 Δ*hopG1* mutant in wild-type Col-0 and in *Atnhr2b* mutant plants at 3 days post-inoculation (dpi) (Fig. 2). As an adapted pathogen of *Arabidopsis,* the wild-type strain *Pst* DC3000 is able to grow in wild-type Col-0 to 10^7^ CFU/cm^2^ at 3 dpi. In contrast, the growth of the *Pst* DC3000Δ*hopG1* was ~ 10-fold lower than the growth of wild-type *Pst* DC3000 in wild-type Col-0. In the *Atnhr2b* mutant plants, the growth of the wild-type *Pst* DC3000 was equivalent to its growth in wild-type Col-0 plants. Interestingly, the *Pst* DC3000Δ*hopG1* mutant grew to higher levels (~ 10^7^ CFU/cm^2^) in *Atnhr2b* mutant plants, and those levels were equivalent to the growth of *Pst* DC3000 in wild-type Col-0. These results suggest that the *Atnhr2b* mutation restores the growth defect in the *PstDC3000*Δ*hopG1* mutant supporting a functional relationship between HopG1 and AtNHR2B.

### The *Pst* DC3000 effector HopG1 interacts with AtNHR2B

**Figure 3:**
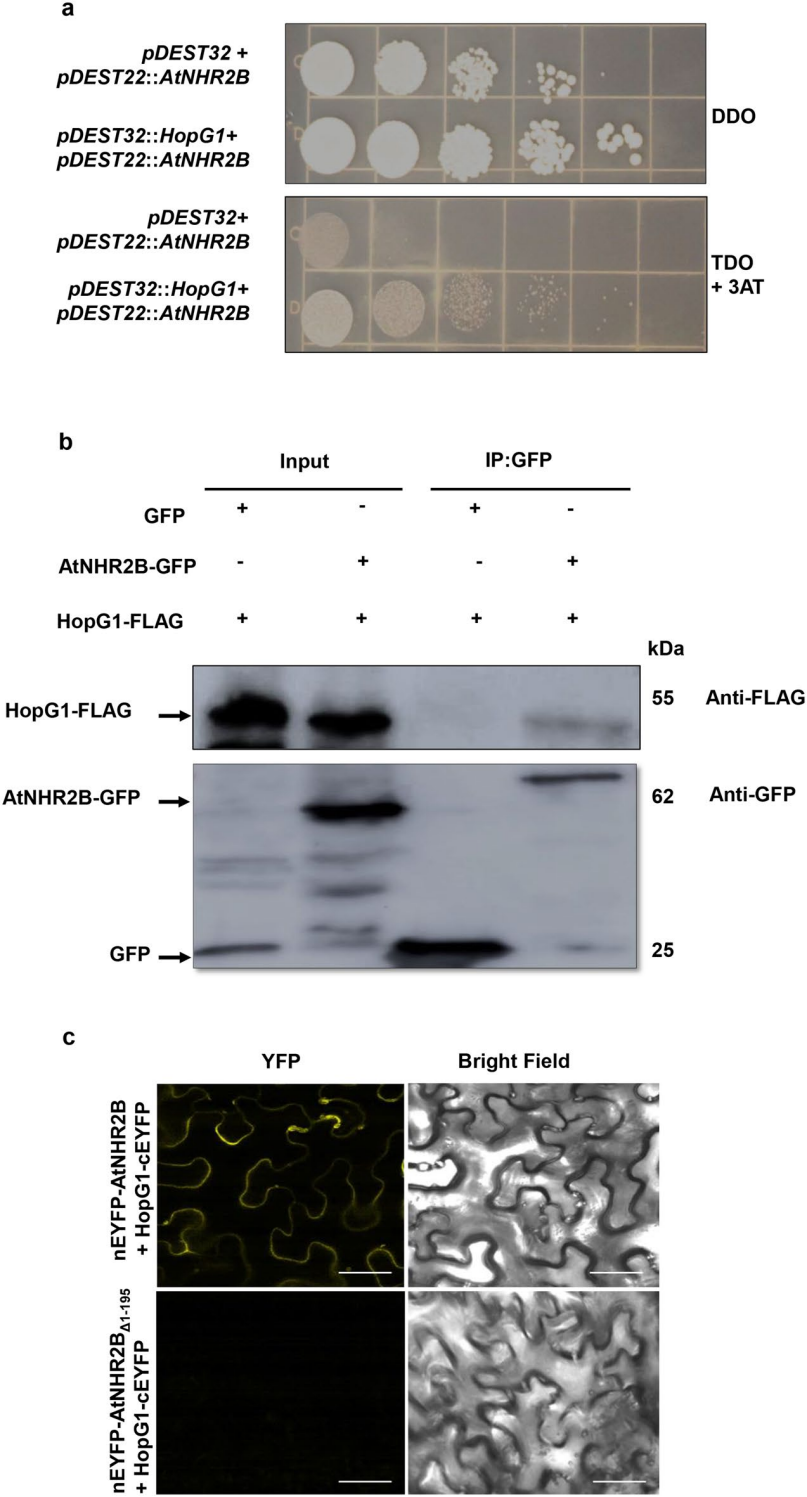
HopG1 interacts with AtNHR2B. (**a**) HopG1 interacts with AtNHR2B in yeast. Yeast strain MaV203 was co-transformed with *pDEST32::HopG1* and *pDEST22:: AtNHR2B*, or *pDEST32* and *pDEST22::AtNHR2B* and transformants were isolated in on Double Drop Out (DDO, SD/-Leu/-Trp) media and transferred to Triple Drop Out (TDO, SD/-His/-Leu/-Trp) + 15 mM 3-Amino-1,2,4-Triazole**.** All the experiments were repeated three times and shown similar results. (**b**) HopG1 and AtNHR2B-GFP interact in planta. *HopG1-FLAG* and *AtNHR2B-GFP*, or *HopG1-FLAG* and *35S-GFP* combinations were transiently co-expressed in 5-week-old *N. benthamiana* plants. Infiltrated leaves were harvested for protein extraction followed by immunoprecipitation using GFP Nanobody/VHH coupled to agarose beads. Immunoprecipitated samples were separated by SDS-PAGE electrophoresis and transferred to a Polyvinylidene difluoride (PVDF) membrane for Western Blot analysis using anti-FLAG and anti-GFP antibodies. (**c**) Bimolecular fluorescence complementation analysis shows the interaction between HopG1 and AtNHR2B in *planta. HopG1* fused to the C-terminal encoding half of *EYFP* (*HopG1- cEYFP*), was transiently co-expressed in *N. benthamiana* with *AtNHR2B* fused to the N-terminal encoding half of *EYFP* (*nEYFP-AtNHR2B*), or with the truncated version AtNHR2B _Δ1–195_ fused to the N-terminal encoding half (*nEFYP-*AtNHR2B _Δ1–195_). At 3 dpi, infiltrated leaves were imaged by laser scanning confocal microscopy using excitation and emission wavelengths of 488 nm and 500–535 nm, respectively. Confocal images were taken at ×63 magnification. Scale bars = 50 µm.

To start dissecting the functional relationship between HopG1 and AtNHR2B, we tested the physical interaction between AtNHR2B and HopG1 with the yeast two- hybrid system using HopG1 as a bait and AtNHR2B as a prey. Yeast co-transformed with *pDEST32::HopG1* and *pDEST22::AtNHR2B* grew on Triple Drop Out (TDO) media (-leu,-trp,-his) containing 15 mM 3-AT indicating the transcriptional activation of histidine biosynthetic genes, as a result of the interaction between HopG1 and AtNHR2B. That interaction is not the result of auto-activation, because yeast transformed with the empty vector *pDEST32* and *pDEST22::AtNHR2B* did not grow on TDO + 15 mM 3-AT (Fig. 3a).

To evaluate the HopG1/AtNHR2B interaction in the appropriate biological context, we transiently co-expressed *HopG1* fused to the FLAG epitope (*HopG1-FLAG*) with AtNHR2B-GFP for co-immunoprecipitation. *HopG1-FLAG* and free *GFP* were also co-infiltrated and used as control. Immunoprecipitation of AtNHR2B-GFP using GFP Nanobody/V_H_H coupled to agarose beads (GFP-Trap^®^ Agarose, Chromotek), co-immunoprecipitated HopG1-FLAG as detected by Western Blot using Anti-FLAG antibodies (Fig. 3b, Supplementary Fig. 3). In contrast, immunoprecipitation of free GFP did not co-immunoprecipitate HopG1-FLAG, demonstrating that the physical interaction between HopG1 and AtNHR2B also occur in planta and it is not an artifact of the GFP tag. We further evaluated the in-situ interaction of AtNHR2B and HopG1 by co-expressing *AtNHR2B* fused to the N-terminal half encoding domain of the enhanced yellow fluorescent protein gene (*nEYFP*), and the *HopG1* fused to the C-terminal half encoding domain of *EYFP* (*cEYFP*) for Bimolecular fluorescence complementation (BiFC) assays^33^. The in-situ interaction between AtNHR2B and HopG1 was revealed by the restoration of the yellow fluorescence and shown to occur in the cytoplasm; importantly, amino acids 1–195 in AtNHR2B are important for the interaction as deletion of the region encoding those amino acids abolishes the interaction with HopG1 (Fig. 3c).

### HopG1 expression causes virulence-related cell death in *N. benthamiana* that is attenuated by AtNHR2B

**Figure 4:**
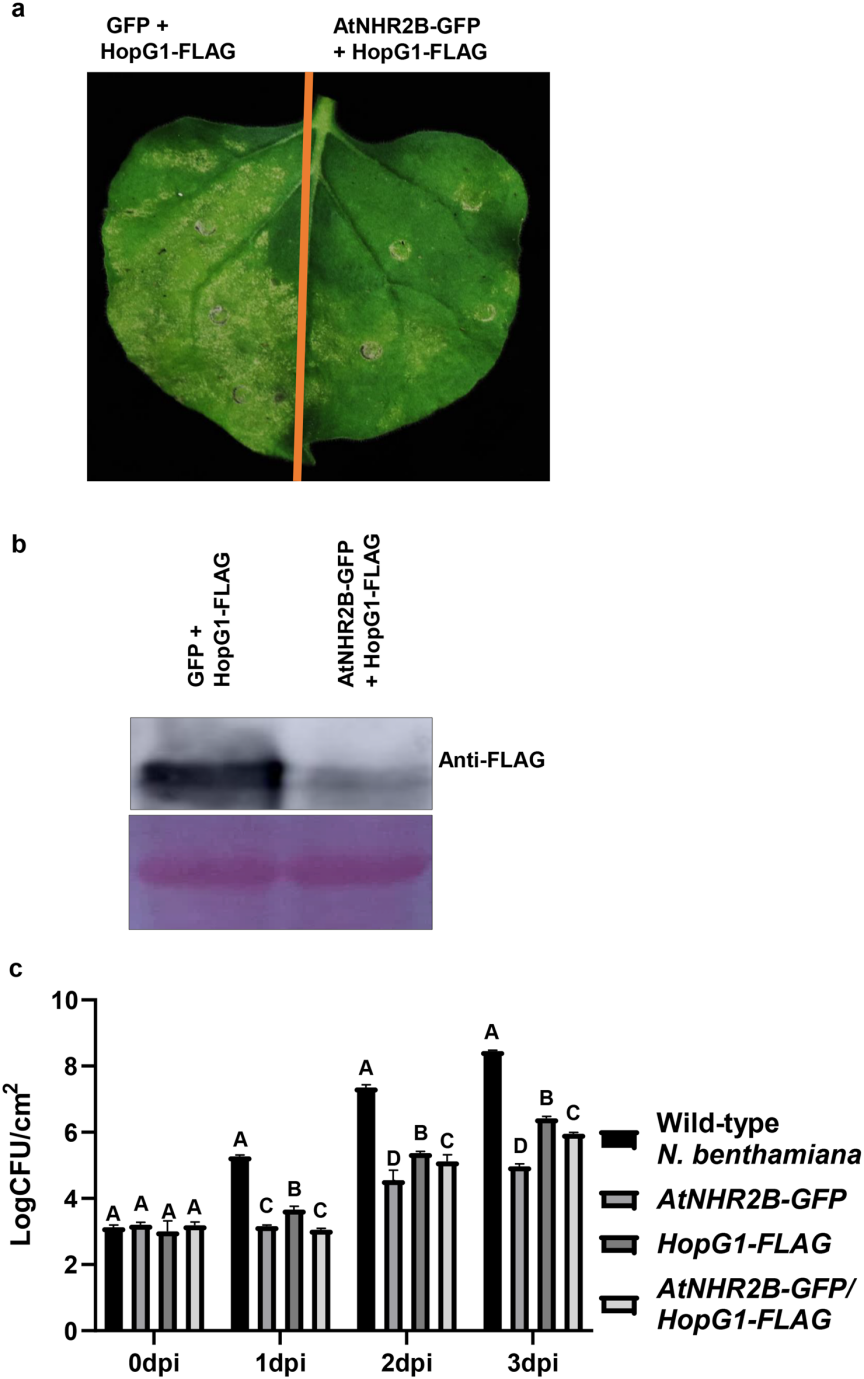
Transient expression of HopG1 in *N. benthamiana* triggers cell death that is attenuated by AtNHR2B. (**a**) Phenotypic evaluation of cell death in *N. benthamiana. HopG1-FLAG* was transiently co- expressed in *N. benthamiana* with *AtNHR2B-GFP* or with *GFP* alone. At 24 hpi, 20 μM DEX was sprayed to induce the expression of *HopG1-FLAG*. Cell death was evaluated at 4 dpi. (**b**) AtNHR2B alters the abundance of HopG1 protein. *HopG1-FLAG* was transiently co- expressed in *N. benthamiana* with *AtNHR2B-GFP* or with *GFP* alone. At 24 hpi, 20 μM DEX was sprayed to induce the expression of *HopG1-FLAG*. Infiltrated leaves were collected at 72 hpi for protein extraction and Western Blot analysis using anti-FLAG antibodies. Ponceau S staining shows equal protein loading. (**c**) Transient expression of *HopG1-FLAG* enhances *Pstab* growth in *N. benthamiana.* Wild-type *N. benthamiana* and *N. benthamiana* plants transiently expressing *AtNHR2B-GFP*, *HopG1-FLAG* and *AtNHR2B-GFP/HopG1-FLAG*, were syringe-inoculated with the adapted bacterial pathogen *Pstab* at a concentration of 1 × 10^4^ CFU/ml. *HopG1-FLAG* and *AtNHR2B-GFP*/*HopG1-FLAG* expressing leaves were sprayed with 20 μM DEX 24 h prior to *Pstab* inoculation. Inoculated leaves were collected at 0, 1, 2 and 3 dpi to enumerate bacterial populations. Bars represent means and standard deviation for bacterial growth in wild-type *N. benthamiana* and in *N. benthamiana* plants transiently expressing *AtNHR2B-GFP*, *HopG1-FLAG* and *AtNHR2B-GFP/HopG1-FLAG.* Different letters above bars represent statistically significant difference in the growth of *Pstab* in wild-type *N. benthamiana* and *N. benthamiana* plants transiently expressing the aforementioned constructs, using a two-way ANOVA and Tukey’s multiple comparison test with P-value ≤ 0.05. The experiments were repeated three times with consistent results.

To further understand how the interaction between HopG1 and AtNHR2B in planta was related with the function of HopG1, we transiently co-expressed *HopG1-FLAG* with free *GFP* or with *AtNHR2B-GFP* in *N. benthamiana.* At 4 days after infiltration, the section of the leaf expressing *HopG1-FLAG* alone (Supplementary Fig. 4a), or co-expressed with *GFP* (Fig. 4a) showed extensive cell death, but that cell death was reduced in the section of the leaf co-expressing *HopG1-FLAG* and *AtNHR2B-GFP* (Fig. 4a, Supplementary Fig. 4a). Similar results were obtained when transiently expressing in *N. benthamiana* another version of HopG1 epitope-tagged with HA (*HopG1-HA*), and when transiently co-expressing *AtNHR2B-GFP* with *HopG1-HA* (Supplementary Fig. 4b). To further understand how AtNHR2B reduced the cell death phenotype caused by HopG1, we evaluated HopG1 protein abundance by Western blot. Tissues co-expressing *HopG1-FLAG* with *AtNHR2B-GFP* had reduced levels of *HopG1-FLAG* in comparison with tissues co-expressing *HopG1-FLAG* with *GFP* (Fig. 4b, Supplementary Fig. 5). Similar results were obtained when transiently expressing in *N. benthamiana HopG1-HA* alone or in combination with *AtNHR2B-GFP* (Supplementary Fig. 6). Co-expression of *AtNHR2B-GFP* with *HopG1-FLAG* did not alter the AtNHR2B-GFP protein abundance (Supplementary Fig. 7). Collectively, these results indicate that HopG1 transiently expressed in *N. benthamiana* induces cell death that is attenuated by AtNHR2B degrading HopG1.

Since the cell death phenotype can be an HR defense response or necrosis associated with virulence, we resolved between those two alternatives by inoculating *N. benthamiana* plants transiently expressing *HopG1-FLAG* with *Pstab* to evaluate its growth at 1, 2 and 3 dpi. For these experiments, we took advantage of a version of *Pstab* expressing the fluorescent protein GFP-TIR^34^ (*Pstab-GFP-TIR*), to be able to discriminate *Pstab* from the *A. tumefaciens* strains used for transient expression. As an adapted pathogen of *N. benthamiana, Pstab-GFP-TIR* progressively grew in wild-type *N. benthamiana* and reached more than 10^8^ CFU/cm^2^ at 3 dpi (Fig. 4c). In contrast, *N. benthamiana* plants that were transiently expressing *AtNHR2B-GFP, HopG1-FLAG* or co-expressing *AtNHR2B-GFP* and *HopG1-FLAG* had significantly lower bacterial populations, in comparison with wild-type plants, likely as a result of *Agrobacterium-*induced PTI (Fig. 4c). Interestingly, among transiently-expressing plants, those expressing *HopG1-FLAG* showed the highest levels of *Pstab-GFP-TIR* populations at 1, 2 and 3 dpi. Moreover, while *Pstab-GFP-TIR* populations at 1 dpi were equivalent between plants transiently expressing *AtNHR2B-GFP* alone or in combination with *HopG1-FLAG,* at 2 and 3 dpi *Pstab-GFP-TIR* populations in plants co-expressing *AtNHR2B-GFP* and *HopG1-FLAG* were intermediate when compared with populations in plants expressing either *AtNHR2B-GFP* or *HopG1-FLAG* alone (Fig. 4c). These results are consistent with the reduction in cell death phenotype and HopG1-FLAG accumulation in *N. benthamiana* plants co-expressing *AtNHR2B-GFP* with *HopG1-FLAG.* Taken together, these results show that HopG1 triggers plant cell death that is not an HR, but either contributes to plant susceptibility by suppressing PTI, enhances pathogen’ virulence or both. Moreover, this virulence activity of HopG1-inducing cell death that promotes pathogen multiplication, is actively attenuated by AtNHR2B.

### Arabidopsis transgenic plants expressing HopG1-FLAG induce mitochondrial ROS-related cell death that is attenuated by AtNHR2B

**Figure 5:**
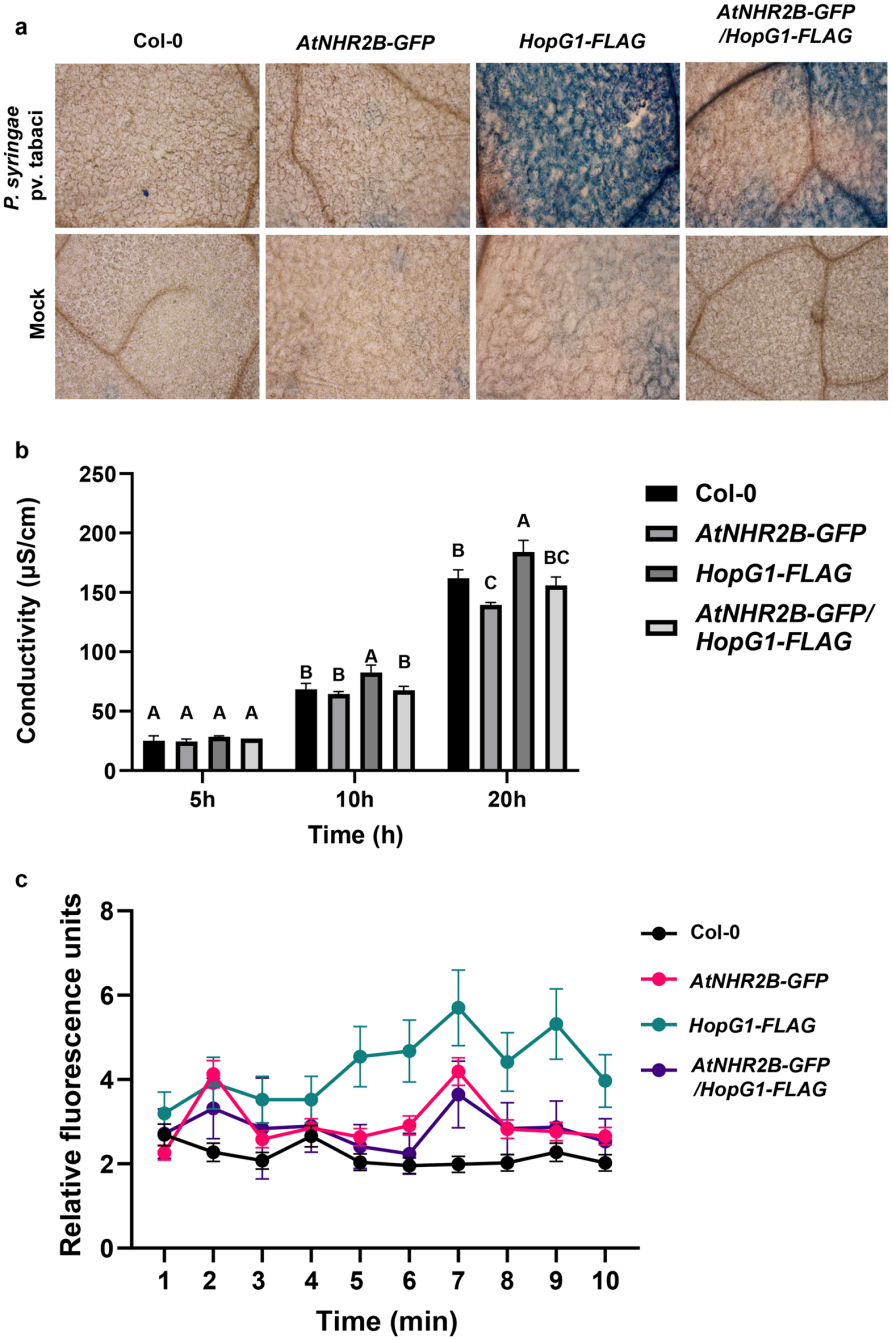
Transgenic expression of HopG1 in Arabidopsis causes cell death that is attenuated by AtNHR2B. (**a**) Cell death phenotype in *HopG1* expressing plants is reduced by expression of *AtNHR2B*. Five-week-old wild-type Col-0, *AtNHR2B-GFP*, *HopG1-FLAG* and *AtNHR2B-GFP*/*HopG1-FLAG* plants were syringe-inoculated with *Pstab* at a concentration of 1 × 10^6^ CFU/mL or infiltrated with water (mock). *HopG1-FLAG* and *AtNHR2B-GFP*/*HopG1-FLAG* plants were sprayed with 20 μM DEX 24 h prior bacterial inoculation. At 24 hpi, treated leaves were detached and stained with 0.05% trypan blue. Images were taken on a light microscope using bright field. (**b**) Ion leakage is enhanced in plants expressing *HopG1*. Five-week-old wild-type Col-0 and transgenic plants expressing *AtNHR2B-GFP, HopG1-FLAG*, and *AtNHR2B-GFP/HopG1-FLAG* were syringe-inoculated with *Pstab* at a concentration of 1 × 10^6^ CFU/ml to evaluate electrolyte leakage at 5, 10, 20 and 24 hpi. *HopG1-FLAG* and *AtNHR2B-GFP*/*HopG1-FLAG* were sprayed with 20 μM DEX 24 h prior to bacterial inoculation. Bars represent means and standard deviation of conductivity values (µS/cm). Different letters above bars represent statistically significant difference in electrolyte leakage among plant genotypes using a one-way ANOVA and Fisher’s multiple comparison test with P-value ≤ 0.05. (**c**) *Arabidopsis* plants expressing HopG1-FLAG generate higher levels of ROS from mitochondrial origin. Wild-type Col-0 and transgenic plants expressing *HopG1-FLAG*, *AtNHR2B-GFP* and *AtNHR2B-GFP/HopG1-FLAG* were flood-inoculated with the non-adapted bacterial pathogen *Pstab* at a concentration of 1 × 10^6^ CFU/ml, or infiltrated with water (mock). *HopG1-FLAG* and *AtNHR2B-GFP*/*HopG1-FLAG* were sprayed with 20 μM DEX 24 h prior bacterial inoculation. Leaf disks were incubated with MitoTracker Red CM-H2XRos and mitochondrial ROS fluorescence was measured using excitation/emission wavelengths of 570 nm and 535 nm, respectively.

To further validate the results observed in *N. benthamiana*, we obtained transgenic *Arabidopsis* lines expressing *HopG1-FLAG* under dexamethasone inducible promoter, and further crossed them with transgenic lines expressing *AtNHR2B-GFP*. Wild-type Col-0 plants and transgenic plants expressing *HopG1-FLAG, AtNHR2B-GFP* and *HopG1-FLAG/AtNHR2B-GFP* were inoculated with the non-adapted pathogen *Pstab* or mock-treated with water to evaluate cell death after staining with Trypan Blue^35^. Inoculation with *Pstab* triggered cell death in transgenic plants expressing *HopG1-FLAG* alone or in combination with *AtNHR2B-GFP,* whereas, no cell death was observed in wild-type Col-0 plants, nor in plants expressing *AtNHR2B-GFP* alone. Importantly, less cell death was observed in transgenic plants co-expressing *HopG1-FLAG* with *AtNHR2B-GFP,* suggesting that AtNHR2B-GFP also attenuates the HopG1-triggered cell death in Arabidopsis (Fig. 5a). We confirmed that to be the case, by using the same plants’ genotypes and pathogen inoculation conditions to quantify cell death by electrolyte leakage at 5-, 10-, and 20-h post-inoculation (hpi). The results showed that as early at 10 hpi, plants expressing *HopG1-FLAG* have the highest levels of electrolyte leakage that were significantly different from the electrolyte leakage levels in Col-0 plants, as well as in plants expressing *AtNHR2B-GFP* and plants co-expressing *HopG1-FLAG* and *AtNHR2B-GFP* (Fig. 5b).

Because cell death phenotypes are regulated by reactive oxygen species (ROS), and mitochondria are one of the source of ROS, it was necessary to evaluate how HopG1 contributes to the production of ROS in the mitochondria. For that purpose, a mitochondria-specific ROS sensor, MitoTracker Red CM-H_2_XRos (ThermoFisher Scientific, Waltham, MA) was used to evaluate mitochondrial ROS produced after pathogen infection. Inoculation of *Pstab* triggered an accumulation of mitochondrial ROS in all plant genotypes. However, the levels of accumulation varied between genotypes. Overall, the lowest levels of mitochondrial ROS were observed in wild-type Col-0 and moderate levels were found in transgenic plants expressing *AtNHR2B-GFP* and co-expressing *HopG1-FLAG* and *AtNH2B-GFP.* Remarkably, at 3 min of the assay, the levels of mitochondrial ROS in plants expressing *HopG1-FLAG* started to increase and those levels peaked at 7 min when they were ~ 3-fold higher than in wild-type Col-0 and ~ 1.5-fold higher than in plants expressing *AtNHR2B-GFP* or co-expressing *AtNHR2B-GFP* and *HopG1-FLAG* (Fig. 5c, Supplementary Table S1). These results showed that the HopG1-mediated cell death is associated with the production of reactive oxygen species in the mitochondria where HopG1 localizes.

### HopG1 interferes with AtNHR2B function to promote disease

**Figure 6:**
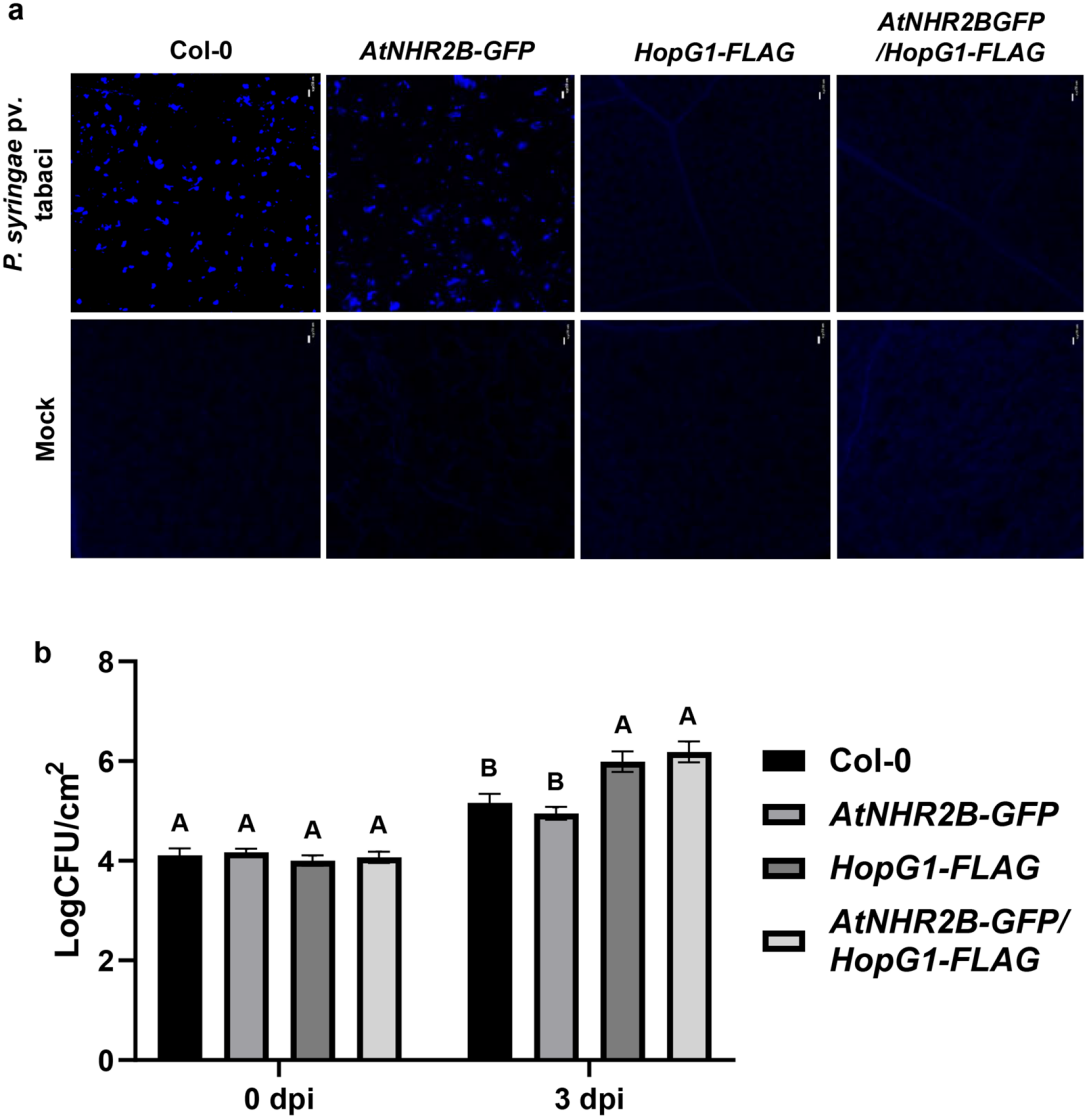
Transgenic expression of HopG1 interferes with AtNHR2B-dependent plant defense responses. (**a**) Plants expressing *HopG1* are deficient in callose deposition. Five-week-old wild-type Col-0, *AtNHR2B-GFP, HopG1-FLAG*, and *AtNHR2B-GFP/HopG1-FLAG* were syringe-inoculated with the non-adapted bacterial pathogen *Pstab* at a concentration of 1 × ^5^/ml. *HopG1-FLAG* and *AtNHR2B-GFP*/*HopG1-FLAG* were sprayed with 20 μM DEX 24 h prior to bacterial inoculation. Inoculated leaves were detached at 24 hpi and stained with 5% aniline blue staining to evaluate callose deposition. Images were taken using a confocal microscope under DAPI filter. Scale bar = 20 μm. (**b**) Transgenic expression of HopG1- contributes to bacterial growth. Wild-type Col-0, *AtNHR2B-GFP, HopG1-FLAG*, and *AtNHR2B-GFP/HopG1-FLAG* were syringe inoculated with *Pstab* at a concentration of 1 × 10^6^ CFU/ml. *HopG1-FLAG* and *AtNHR2B-GFP*/*HopG1-FLAG* were sprayed with 20 μM DEX 24 h prior bacterial inoculation. Leaf samples were collected at 0 and 3 dpi. Bars represent means of CFU/cm^2^. Different letters above bars represent statistically significant difference in bacterial growth among plant genotypes using a one-way ANOVA and Tukey’s multiple comparison test with P-value ≤ 0.05. All above experiments were repeated three times with similar results.

Our results demonstrating an interaction between HopG1 and AtNHR2B and a possible interplay between both proteins, prompted us to further investigate the antagonism between HopG1 and AtNHR2B. HopG1 was previously shown to interfere with callose deposition when *HopG1-HA* expressing plants were inoculated with the *Pst* DC3000 *hrcC* mutant^24^. In our assays, we used *Pstab* that also triggers PTI in Arabidopsis as demonstrated by the callose deposits in the wild-type Col-0 plants and in transgenic Arabidopsis plants expressing *AtNHR2B-GFP* (Fig. 6a). As previously reported, transgenic plants expressing *HopG1-FLAG* were devoid of callose deposits and were comparable to mock-treated plants. Similarly, plants co-expressing *HopG1-FLAG* and *AtNHR2B-GFP* were also devoid of callose deposits suggesting that AtNHR2B is unable to interfere with the PTI-suppressing activities of HopG1 (Fig. 6a).

Consistent with the activities of HopG1 suppressing PTI and the inability of AtNHR2B to interfere with HopG1-mediated PTI suppression, transgenic plants expressing *HopG1-FLAG* alone or in combination with AtNHR2B-GFP supported 10-fold higher growth of *Pstab* at 3 dpi in comparison with *Pstab* growth in Col-0, or in plants expressing *AtNHR2B-GFP* (Fig. 6b). Taken together, these results demonstrate that in Arabidopsis, HopG1 interferes with plant defense responses even when AtNHR2B-GFP is overexpressed. Moreover, the increased bacterial growth and lack of callose deposition, suggest that similar to the results obtained in *N. benthamiana,* the HopG1-mediated cell death is not related to the HR but to a virulence mechanism related to HopG1 function.

## Discussion

*Pst* DC3000 deploys a plethora of effectors into the plant cell to interfere with plant defense responses, alter cellular processes and promote bacterial parasitism^6,7^. The *Pst* DC3000 T3E HopG1 appears to be of paramount importance in the pathogenicity of *Pst* DC3000 by being one of five effectors constituting the minimal repertoire that makes *Pst* DC3000Δ*hopQ1-1* pathogenic in *N. benthamiana*^4,23^. Previous studies also demonstrated that HopG1 suppresses the HR in *N. benthamiana* based on experimental data showing that only the *Pst* DC3000 *hopG1* mutant triggered the HR, whereas the wild type *Pst* DC3000, or the *Pst *DC3000 *hopG1* (*phopG1*) complementation strain  did not^26^. The elicitation of the HR by the *Pst* DC3000 *hopG1* mutant, suggests that the absence of HopG1 uncovered the HR eliciting activity of other effectors. In contrast, the presence of HopG1 in wild type *Pst* DC3000, and in the *Pst *DC3000 *hopG1* (*phopG1*) complementation strain, suppresses the HR. Furthermore, transient expression of the cell death inducer *BAX1*^36^ in *N. benthamiana* triggered cell death, as expected, but this cell death was not observed when *BAX1* was transiently co-expressed with *HopG1*^26^.

In addition to suppressing the HR, transgenic expression of *HopG1-HA* in Arabidopsis also led to suppression of PTI, observed as a reduction in callose deposition after infiltration with the Flg 21 peptide, or after inoculation with the non-pathogen *Pseudomonas fluorescens*^24^. We confirmed these results by showing that Arabidopsis plants expressing *HopG1-FLAG* and inoculated with the non-adapted pathogen *Pstab* are also devoid of callose deposits*.*

Other previous observations on the virulence function of HopG1, revealed its role in the remodeling of the cytoskeleton demonstrated to occur through the interaction of HopG1 with the mitochondrial-localized kinesin motor protein^27^. The mitochondrial localization of HopG1 previously led to the hypothesis that HopG1 alters mitochondrial function, and in support of that hypothesis, Arabidopsis plants expressing *HopG1-HA* showed reduced oxygen consumption and enhanced ROS levels^24^. However, that study used the ROS sensitive probe H2DCFDA (2′-7′-dichlorodihydrofluorescein), that does not discriminate among the multiple sources of ROS. In this study, we used a mitochondria-specific fluorogenic ROS sensor and defined more precisely that the higher levels of ROS in *HopG1-*FLAG transgenic lines are from mitochondrial origin. Thus, our results provide stronger evidence that *HopG1-FLAG* expression and localization to mitochondria actually induces the production of mitochondrial ROS that likely activate a cell death program.

In this study, we uncovered that indeed HopG1 induces necrotic cell death by transient expression in *N. benthamiana* and by stable expression in Arabidopsis. Previously, *Agrobacterium-*mediated transient expression of *Pst* DC3000 *HopG1* in 59 plant accessions that included *N. benthamiana,* revealed that this effector caused cell death in a limited number of plants (ca 5)^37^. Such cell death phenotype was explained as a recognition by the plant (HR) given that the effector was from a non-adapted pathogen^36^. However, a *Pst* DC3000 strain deleted of all effectors and only harboring *HopG1* failed to elicit the HR in *N. benthamiana*^38^, demonstrating that HopG1 by itself is unable to cause the HR. Our data supports the latter findings and further demonstrates that actually, the cell-death phenotype observed is the result of the virulence function of HopG1.

The cell death phenotypes that effectors trigger can be difficult to assess as they might correspond to completely opposite outcomes: the HR, a plant defense response that restricts pathogen proliferation, or necrosis, a virulence function of the pathogen that sustains pathogen proliferation. Thus, to resolve if the HopG1-dependent cell death was HR or virulence-related necrosis, we inoculated the *HopG1-FLAG*-expressing *N. benthamiana* plants with its adapted pathogen *Pstab* to evaluate bacterial growth as evidence of bacterial virulence. We showed that plants expressing *HopG1-FLAG* sustained higher levels of *Pstab* in comparison with plants not expressing *HopG1-FLAG.*Similarly, transgenic Arabidopsis plants expressing *HopG1-FLAG* enabled the growth of *Pstab* which is a non-adapted pathogen in Arabidopsis. Because *Pstab* do not encode *HopG1*^39^, and HopG1 in very important in the pathogenesis of *Pst* DC3000, these results demonstrate that transient or stable expression of *HopG1-FLAG* in plants supplements the deficiency of *HopG1* in *Pstab* making it a better pathogen in both its host plant as well as in a non-host plant.

We also uncovered a functional relationship between HopG1 and AtNHR2B based on the following lines of evidence: (1) *Atnhr2b* mutation restores the growth deficiency of the *Pst* DC3000 Δ*hopG1* mutant; (2) HopG1 directly interacts with AtNHR2B in yeast and in planta; (3) AtNHR2B suppresses the necrotic cell death induced by HopG1 and (4) AtNHR2B degrades HopG1. The results indicate that HopG1 and AtNHR2B counteract each other with HopG1 inducing necrotic cell death and AtNHR2B attenuating HopG1-induced necrosis. This interplay is clear with the results showing that in *N. benthamiana* transient expression of *HopG1-FLAG* enhances the virulence of *Pstab* increasing its populations, whereas co-expression of *HopG1-FLAG* and *AtNHR2B-GFP* in *N. benthamiana* effectively reduces *Pstab* populations. Intriguingly, in Arabidopsis, HopG1-mediated cell-death related phenotypes are attenuated by AtNHR2B, while callose deposition and *Pstab* growth are not. Altogether, these results would indicate a specific function of AtNHR2B attenuating necrotic cell death possibly through targeted degradation of HopG1. More studies are needed to fully understand the interplay between HopG1 and AtNHR2B.

Collectively, our results and the results of others highlight that HopG1 is a multi-faceted protein that can suppress the HR, interfere with plant defense responses and cause necrotic cell death associated with chlorotic symptoms. All these phenotypes associated with HopG1 highlight its function early in infection, suppressing immune responses, and later in infection triggering necrotic cell death. The functions of HopG1 are consistent with the hemi-biotrophic lifestyle of *Pst* DC3000 that combines an early biotrophic phase suppressing cell death responses with a late necrotrophic phase inducing cell death.

## Materials and methods

### Bacterial strains

Wild-type *Pstab, Pst* DC3000 and *Pst* DC3000Δ*hopG1* mutant were grown on King’s B (KB) medium supplemented with rifampicin (25 μg/ml). *Agrobacterium tumefaciens* strains were grown in Luria‐Bertani (LB) medium supplemented with rifampicin (25 μg/ml) and kanamycin (50 µg/ml).

*Pstab-GFP-TIR* was generated by introducing the plasmid *pGFP-TIR*^34^ into *Pstab* by electroporation. *Pstab-GFP-TIR* was grown on KB medium supplemented with gentamycin (20 µg/ml). All the strains were grown at 28 °C. Bacterial strains used in this study are listed in Supplementary Table S2.

### Plant materials and growth conditions

*Arabidopsis thaliana* seeds were planted in soil for two weeks, transplanted to individual pots and grown for four more weeks. Plants were grown in growth chambers at 21 °C with an 8/16 h light/dark cycles. *Nicotiana benthamiana* seeds were sown in soil, transplanted after 2 weeks and grown for 4 weeks under growth chamber conditions at 25 °C with a 10/14 h light/dark cycle.

Transgenic lines expressing *HopG1-FLAG* under the expression of the glucocorticoid promoter were obtained from Dr. Jim Alfano (University of Nebraska, Lincoln). *HopG1-FLAG* plants were crossed with lines expressing *AtNHR2B-GFP* to generate *AtNHR2B-GFP/HopG1-FLAG* transgenic lines.

This research was conducted in compliance with all relevant institutional, national, and international guidelines and legislation for handling of plant materials.

### Plasmid constructs

pENTR/SD:*HopG1*^38^ was transferred into the yeast bait vector *pDEST32* through an LR reaction of Gateway Cloning (Thermo Fisher Scientific, Waltham, MA) to generate a transcriptional fusion to the GAL4-DNA binding domain. *AtNHR2B* in the entry vector *pDONR201* was cloned into the yeast prey vector *pDEST22* to generate a fusion to the GAL4 activation domain.

### Transient expression in *N. benthamiana*

*Agrobacterium tumefaciens* GV2260 harboring constructs of interest were induced and infiltrated into fully expanded leaves of 3-week old *N. benthamiana* plants using a needle-less syringe as previously described^31^. Infiltrated leaves were used to visualize protein localization or in situ protein–protein interaction by laser scanning fluorescence microscopy, or to identify proteins of interest by Western blot.

### Western blot

*Nicotiana benthamiana* transiently expressing proteins of interest were collected in liquid nitrogen. Tissue was ground in liquid nitrogen and homogenized with protein extraction buffer (50 mM Hepes-pH 7.5, 250 mM sucrose, 10 mM EDTA, 5% glycerol, 50 mM sodium pyrophosphate, 25 mM sodium fluoride, 1 mM sodium molybdate, 3 Mm DTT, 1 mM PMSF and plant protease inhibitor cocktail) (Thermo Scientific)^40^. *Arabidopsis* plants expressing *AtNHR2B-GFP* were harvested in liquid nitrogen and homogenized with protein extraction buffer (100 mM Tris–HCl, pH 7.5, 150 mM NaCl, 1 mM EDTA, 10 mM MgCl_2_ 0.20% NP40, 0.1% SDS, 5 mM DTT, 10% glycerol, 1 mM phenylmethylsulfonyl fluoride (PMSF) and plant protease inhibitor cocktail) (Thermo Scientific).

The concentrations of protein in the supernatant were determined by using the Bio-Rad protein assay reagent (Bio-Rad). Samples, typically 30–50 mg, were separated on 10% acrylamide containing SDS-PAGE gels (mini protean; Bio-Rad) and transferred to polyvinylidene fluoride membrane (GE Healthcare). Membranes were incubated with appropriate antibodies: anti-GFP (1:2,000 dilution; Invitrogen), Anti-FLAG (A5892, Sigma) and anti-HA antibody (H6908, Sigma), were used at dilution of 1:1,000. Rabbit antisera (GE Healthcare) was used as secondary antibody with 1:5,000 dilution. Chemiluminescent detection was done by using the ImageQuant™ LS-500 image system (GE Healthcare).

### Live-cell imaging

*Nicotiana benthamiana* plants transiently expressing *AtNHR2B-GFP*, co-expressing *AtNHR2B-nEYFP* with *HopG1-cEYFP,* or *AtNHR2B*_*Δ1-195*_*nEYP with HopG1-cEYFP* were used to evaluate *AtNHR2B-GFP* localization, or *AtNHR2B/HopG1* interaction by bimolecular fluorescence complementation (BiFC), respectively. GFP and YFP signals were imaged on a Leica SP2 or Leica Stellaris 8 Laser Scanning Confocal Microscope (Leica Microsystems, Buffalo Grove, IL) at 48hpi. Arabidopsis plants expressing *AtNHR2B-GFP* were also evaluated by laser scanning fluorescence microscopy. GFP and YFP fluorescence was imaged using excitation wavelength of 488 nm and an emission wavelength of 500 to 535 nm.

### Bacterial inoculation into *Arabidopsis thaliana* plants

Five-week-old *Arabidopsis thaliana* plants, genotypes wild-type Col-0, *Atnhr2b* mutant, or expressing *AtNHR2B-GFP, HopG1-FLAG* and *AtNHR2B-GFP/HopG1-FLAG* were syringe-inoculated with *PstDC3000, PstDC3000 (ΔhopG1*) or *Pstab* at different concentrations depending on the experiment.

### Bacterial multiplication assays

Leaf disks (0.5 cm^2^) from inoculated plants were collected, serially-diluted and plated as previously described^31^. Each experiment was repeated three times.

### Yeast two-hybrid assay

The yeast strain Mav203 was grown in YPDA at 30 °C overnight with constant shaking. The OD_600_ of overnight grown cultures was measured, diluted to an OD_600_ of 0.4 in a final volume of 50 ml of YPAD and grown for additional 3 h. Yeast cells in mid-log phase were co-transformed with pDEST32/pDEST22::*AtNHR2B* or pDEST32::*HopG1*/ pDEST22::*AtNHR2B* using the Frozen-EZ Yeast Transformation II Kit (Zymo Research, Irvine, CA). Transformed yeast cells were plated on Double Drop Out (DDO, SD/-Leu/-Trp) selection plates and grown at 30 °C for 4 days. Single colonies were picked from the plates and cultured in 15 mL DDO broth at 30 °C overnight. The overnight culture was diluted to an OD_600_ of 0.2 and plated on triple dropout medium (TDO, SD/-His/-Leu/-Trp) containing 15 mM 3-Amino-1,2,4-Triazole (3-AT) and grown at 30 °C for 4 days.

### Co-immunoprecipitation

Four-week-old *N. benthamiana* plants were co-infiltrated with two strains of *A. tumefaciens* strains harboring *AtNHR2B-GFP* and *HopG1-FLAG*, or *A. tumefaciens* strains harboring *AtNHR2B-GFP* and 35S-*GFP.* Infiltrated leaves were collected at 2 dpi and tissue, ground in liquid nitrogen and homogenized in 1 mL of co-immunoprecipitation extraction buffer (100 mM Tris–HCl, pH 7.5, 150 mM NaCl, 1 mM EDTA, 10 mM MgCl2, 10% Glycerol, 0.2% Nonidet P-40, 1 mM PMSF, 5 mM DTT, 1X Proteinase inhibitor cocktail (Sigma Aldrich, St. Louis, MO). Protein extracts were incubated for 30 min on ice and centrifuged at 4 °C for 30 min at 13,000 rpm. Supernatants containing extracted proteins were collected in a pre-chilled 1 ml Eppendorf tube. Protein concentration was measured by Bradford Assay (BioRad, Hercules, CA) and protein expression in input samples was confirmed by Western blot as described above.

One milligram of total protein extract was mixed with 25 µl of GFP Nanobody/V_H_H coupled to agarose beads (GFP-Trap^®^ Agarose, Chromotek) and incubated for 3 h and 30 min at 4 °C with end to end rocking. After incubation, protein complexes bound to beads were washed once with 1 × TBS buffer (50 mM Tris–HCl, 150 mM NaCl, pH 7.5) and twice with 1 × TBS buffer (50 mM Tris–HCl, 500 mM NaCl, pH 7.5). Protein complexes bound to the beads were eluted in 2 × SDS protein loading buffer, loaded and ran into an SDS-PAGE gel and transferred to PVDF membranes. Proteins were detected by Western Blot as described above.

### Cell death assay

Five-week old wild-type Col-0, *AtNHR2B-GFP*, *HopG1-FLAG* and *AtNHR2B-GFP*/*HopG1-FLAG* plants were syringe-inoculated with *Pstab* at OD_600_ = 0.02 (1 × 10^6^ CFU/ml). Control plants were inoculated with water only. At 24 hpi, six to nine inoculated leaves were detached from six independent plants for each genotype. Collected leaves were stained with 0.05% trypan blue for 45 min at room temperature and washed twice with PBS^35^. Images were taken on a light microscope using bright field.

### Callose deposition

Five-week old wild-type Col-0, *AtNHR2B-GFP*, *HopG1-FLAG* and *HopG1-FLAG*/*AtNHR2B-GFP* were sprayed with 20 µM dexamethasone to induce expression of *HopG1-FLAG.* At 24 h after dexamethasone treatment, plants were syringe-inoculated with *Pstab* at OD_600_ = 0.02 (1 × 10^6^ CFU/ml). Ten leaves from six independent plants for each genotype and inoculated with *Pstab* or infiltrated with water were detached after 24 hpi and stained with 5% aniline blue to visualize callose deposits. Images were taken by Nikon 90i upright scanning laser confocal microscope (Nikon) using a DAPI (4′,6-diamidino-2-phenylindole) filter with excitation wavelength of 405 nm and an emission wavelength of 450–510 nm.

### Electrolyte leakage assay

Five- week old wild-type Col-0, *AtNHR2B-GFP*, *HopG1-FLAG* and *AtNHR2B-GFP*/*HopG1-FLAG* plants were induced by dexamethasone prior to inoculation with *Pstab* at OD_600_ = 0.02 (1 × 10^6^ CFU/ml). Twelve leaves were collected immediately after infiltration and 0.5 cm^2^ leaf disks were excise and rinsed with 50 ml for 30 min, to wash ions released during disc excision. After 30 min, water was replaced with 12 ml of fresh deionized water and conductivity was measured at 5, 10, and 20 hpi using the Orion Star A215 conductivity cell (013005MD) (Thermo Fisher Scientific).

### Mitochondrial ROS production

Five-week old wild-type Col-0, *AtNHR2B-GFP*, *HopG1-FLAG* and *AtNHR2B-GFP*/*HopG1-FLAG* plants grown on soil were sprayed with a 20 µM dexamethasone solution supplemented with 0.01% silwet, 12 h before inoculation. Leaf disks cut out using a 1.2 cm^2^ core-borer and harvested tissue was transferred to clear bottom plates for fluorometric analysis and submerged in *Pstab* inoculum at a final concentration of 1 × 10^7^ CFU/ml. For mock-treatment, plants were submerged in water. Two hours after inoculation with either *Pstab* mock-treatment, MitoTracker Red CM-H_2_XRos (ThermoFisher Scientific, Waltham, MA) was added at a final concentration of 0.005 mM and incubated for 10 min before taking the first reading. Fluorescence was measured with an excitation wavelength of 570 nm and an emission wavelength of 535 nm on BioTek luminescence microplate reader.

## Supplementary Information

Supplementary Information.
