# Supplementary material for: The Pseudomonas syringae type III effector HopG1 triggers necrotic cell death that is attenuated by AtNHR2B
Source: Sci Rep. 2022 Mar 30;12:5388. doi: 10.1038/s41598-022-09335-1 (PMC8967837; doi:10.1038/s41598-022-09335-1)
Supplement: Supplementary file 1 — Supplementary Information. [file 41598_2022_9335_MOESM1_ESM.pdf]

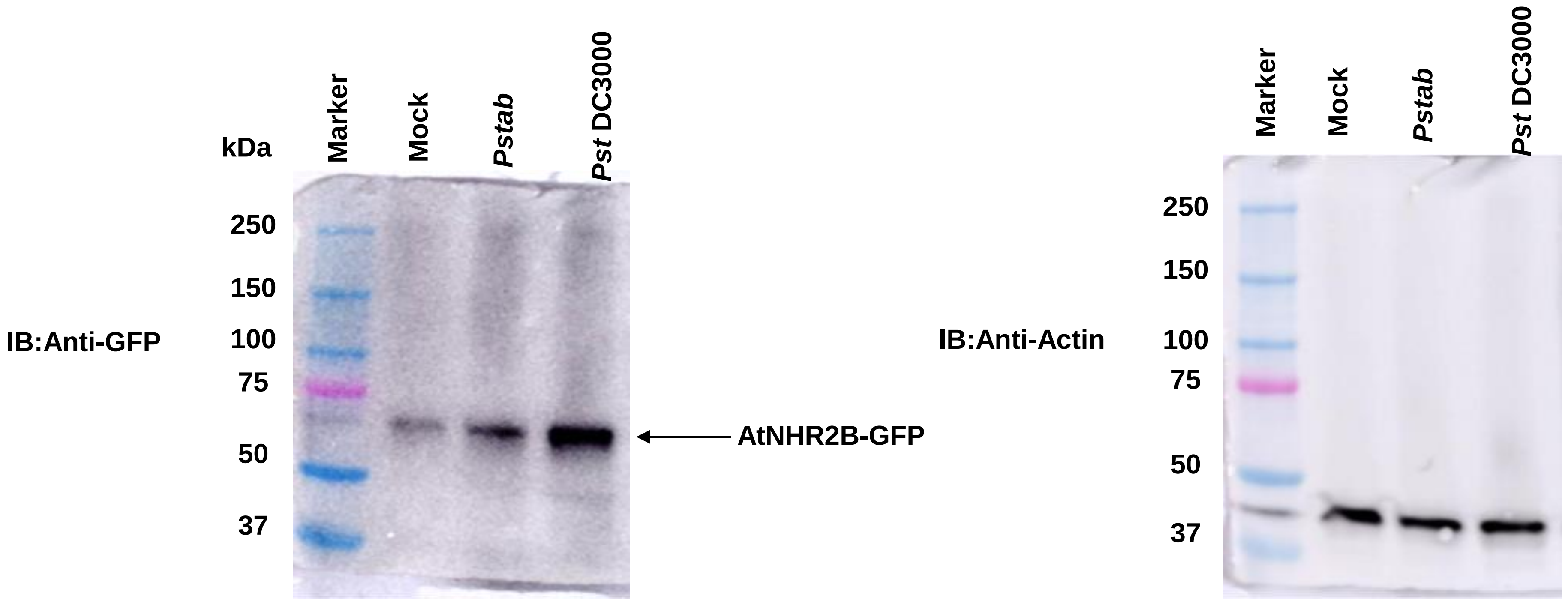


**Figure S 1.** The host adapted pathogen of *Nicotiana benthamiana Pstab* alters AtNHR2B-GFP protein abundance in tobacco plants. *N. benthamiana* plants transiently expressing *AtNHR2B-GFP* were infiltrated with water (mock), or inoculated with either the adapted pathogen *Pstab* or the non-adapted pathogen *Pst* DC3000. Treated leaf samples were collected after 24 hpi to evaluate protein abundance by Western blot using anti-GFP and anti-actin antibodies. Original picture of Western blot results shown in Figure 1c (top and bottom), before cropping

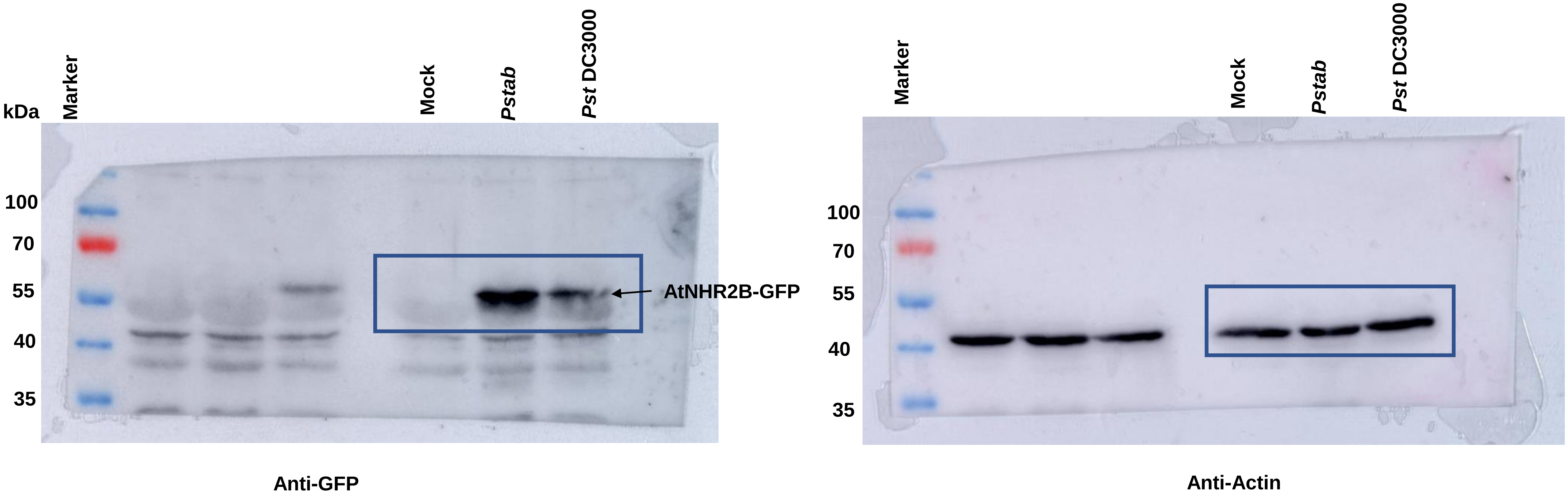


**Fig S2.** The host adapted pathogen of *Arabidopsis, Pst* DC3000, alters AtNHR2B-GFP protein abundance in *Arabidopsis* plants. *A. thaliana* transgenic plants expressing *AtNHR2B-GFP* were infiltrated with water (mock), or inoculated with either the non-adapted pathogen *Pstab* ,or the adapted pathogen *Pst* DC3000*.* Treated leaf samples were collected after 24 hpi to quantify protein abundance by Western blot using anti-GFP and anti-Actin antibodies. Original picture of Western blot results shown in Figure 1d (top and bottom), before cropping.

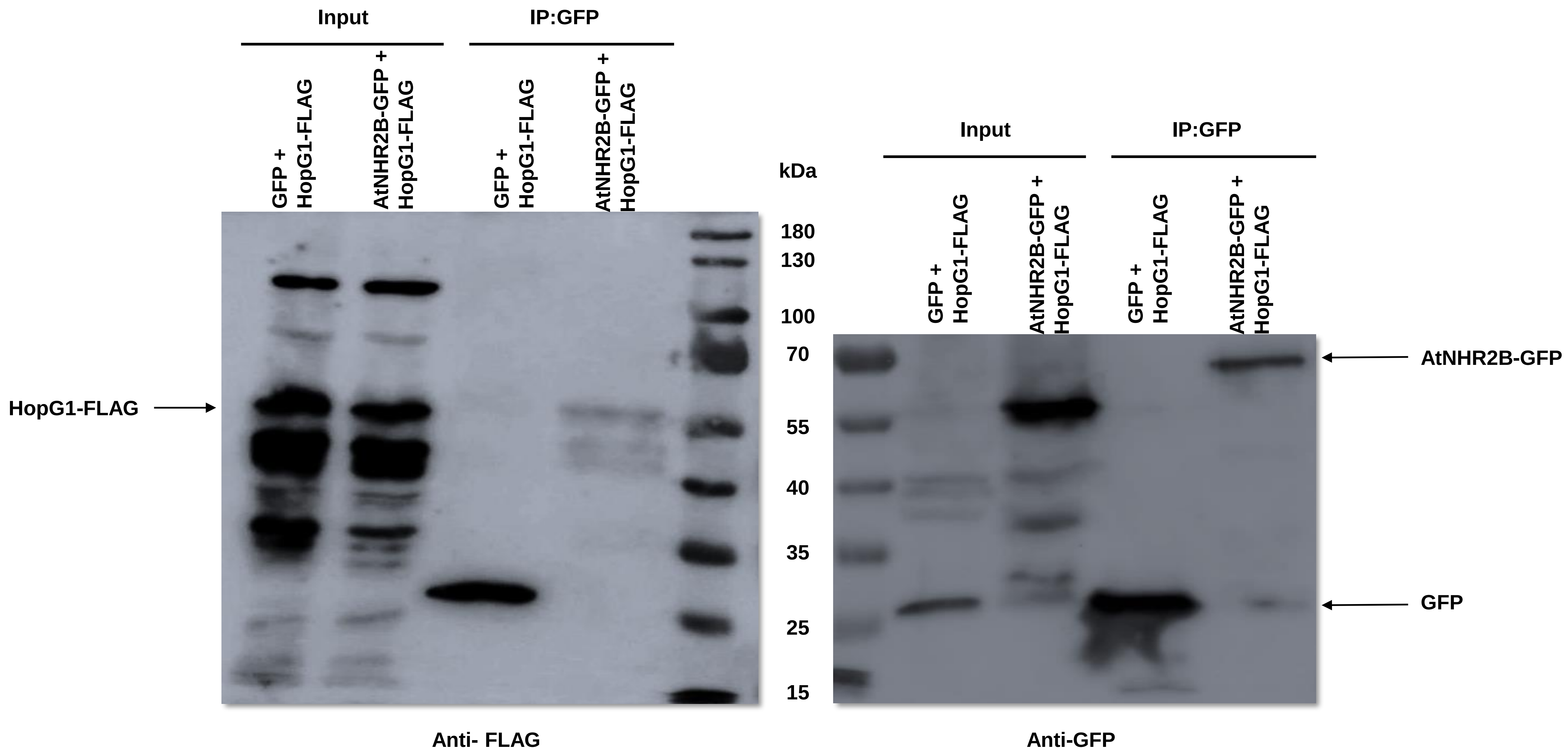


**Fig S3.** HopG1 interacts with AtNHR2B. *Agrobacterium tumefaciens* harboring constructs *HopG1-FLAG, AtNHR2B-GFP*, or *35S-GFP* were co-infiltrated into five-week-old *N. benthamiana* plants, in the combinations shown, for transient expression. Infiltrated leaves were harvested for protein extraction followed by immunoprecipitation using GFP Nanobody/VHH coupled to agarose beads. Immunoprecipitated samples were separated by SDS-PAGE electrophoresis and transferred to a Polyvinylidene difluoride (PVDF) membrane for Western Blot analysis using anti-FLAG (left) and anti-GFP antibodies (right). Original picture of Western blot results shown in Figure 3 before cropping and arrangement. Right-side membrane was cut before incubation with anti- GFP antibodies.

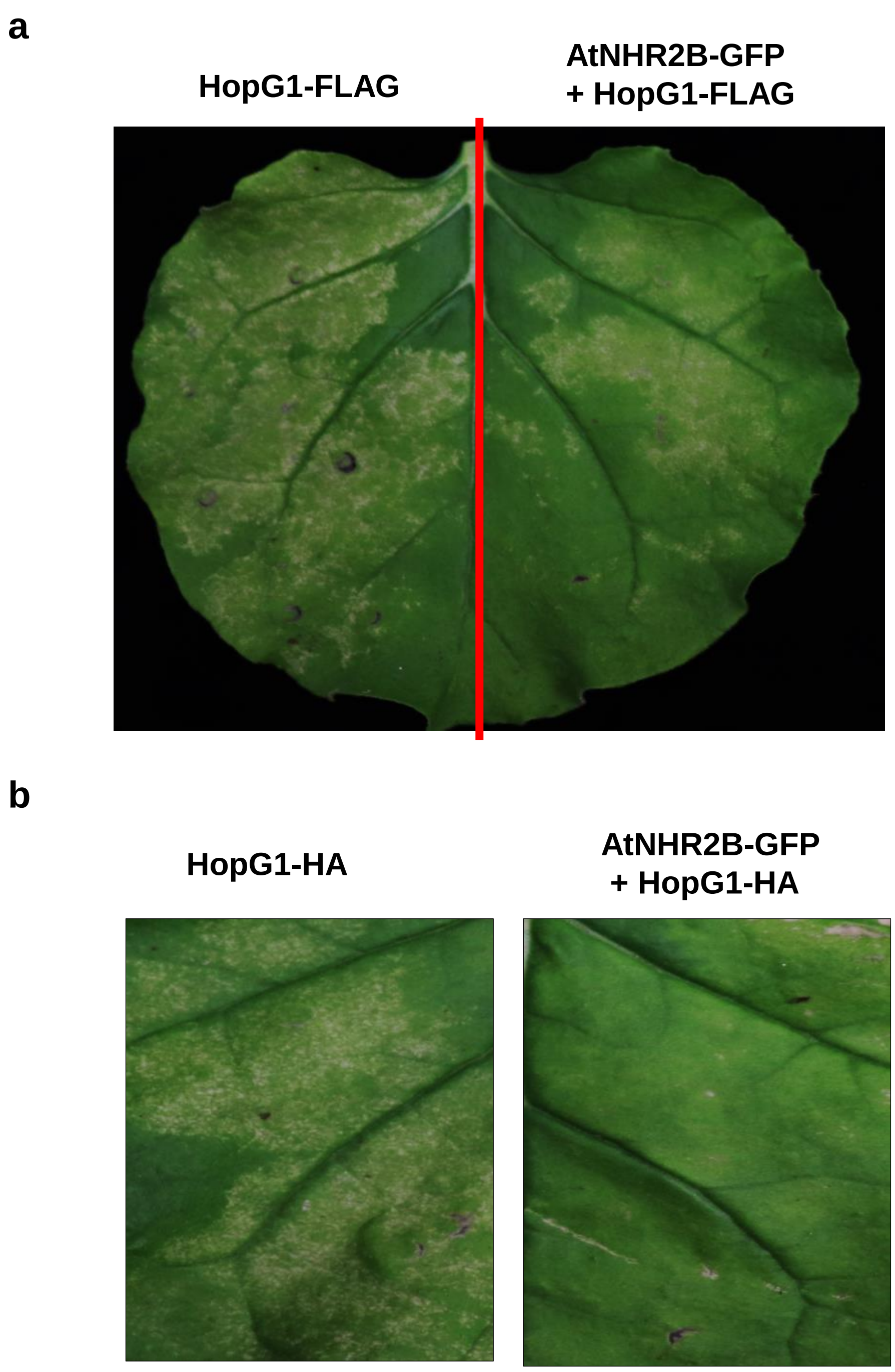


**Figure S 4.** HopG1 triggers cell death in *N. benthamiana* that is attenuated by AtNHR2B. (a) *Agrobacterium tumefaciens* harboring constructs *HopG1-FLAG* and *AtNHR2B-GFP* were infiltrated, alone or in combination, into five-week-old *N. benthamiana* plants for transient expression. b) *Agrobacterium tumefaciens* harboring constructs *HopG1-HA* and *AtNHR2B-GFP* were infiltrated, alone or in combination, into five-week-old *N. benthamiana* plants for transient expression. Cell death was evaluated at 4 dpi.

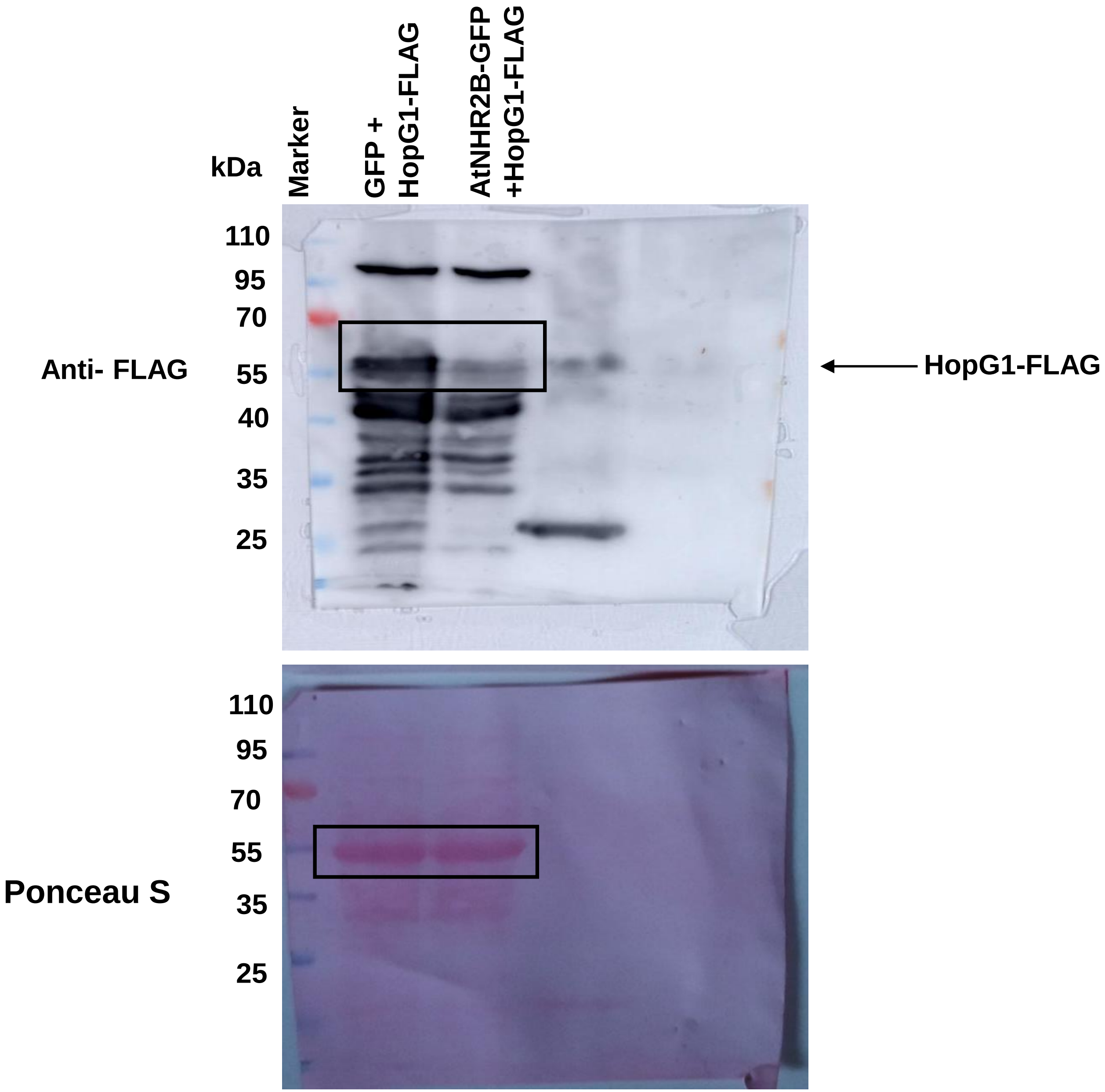


**Figure S 5.** AtNHR2B alters the abundance of HopG1 protein. *HopG1-FLAG* was transiently co- expressed in *N. benthamiana* with *AtNHR2B-GFP* or with *GFP* alone. At 24 hpi, 20μM DEX was sprayed to induce the expression of *HopG1-FLAG*. Inoculated leaves were collected 72 hpi for protein extraction and Western Blot analysis using anti-FLAG antibodies. Ponceau S staining showed equal protein loading. Original picture of the Western blot results shown in Fig 4b before cropping.

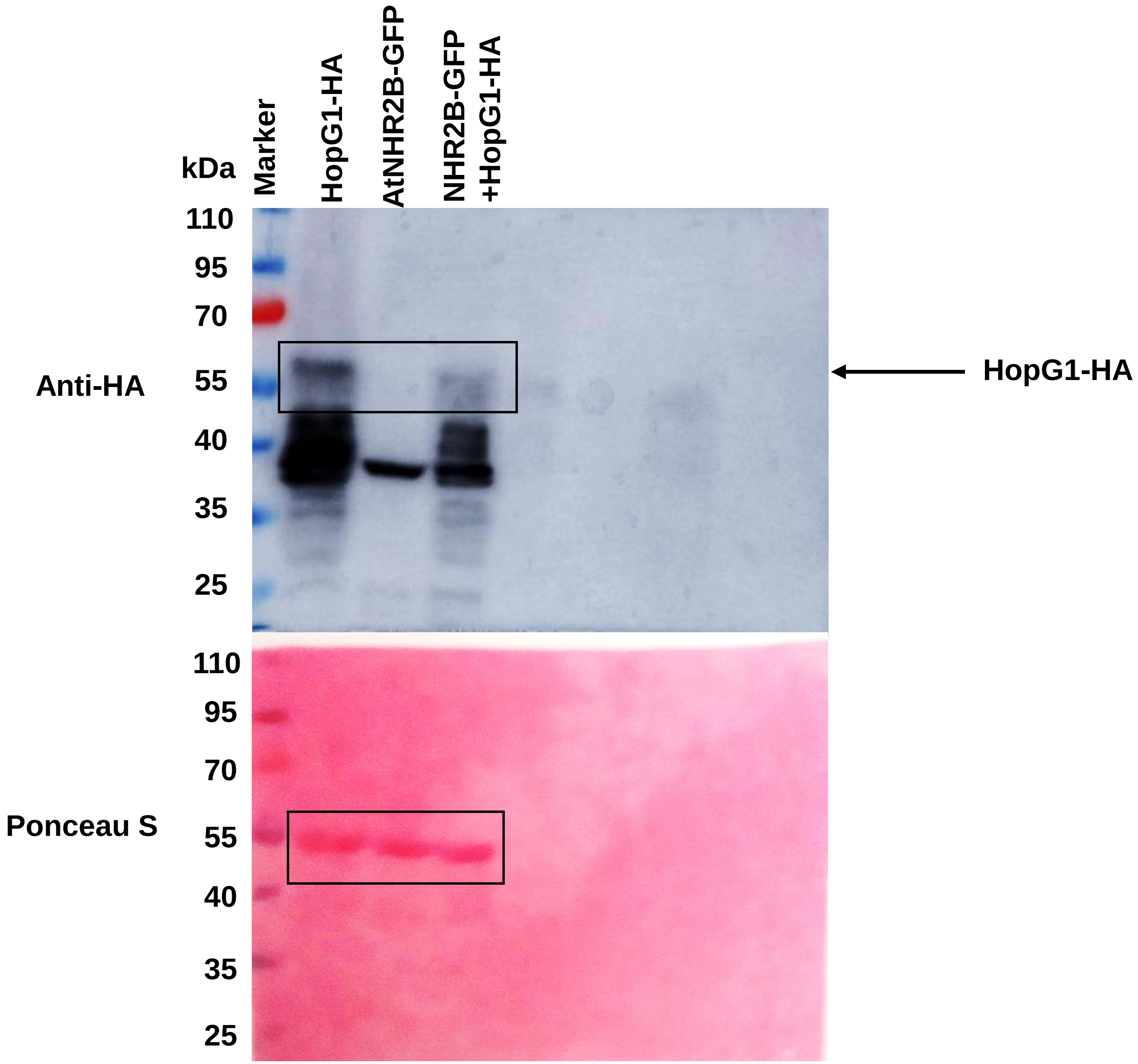


**Figure S 6.** AtNHR2B alters the abundance of HopG1 protein. *HopG1-HA* was transiently co- expressed in *N. benthamiana* with *AtNHR2B-GFP* or with *GFP* alone. Infiltrated leaves were collected 72 hpi for protein extraction and Western Blot analysis using anti-FLAG antibodies. Ponceau S staining showed equal protein loading.

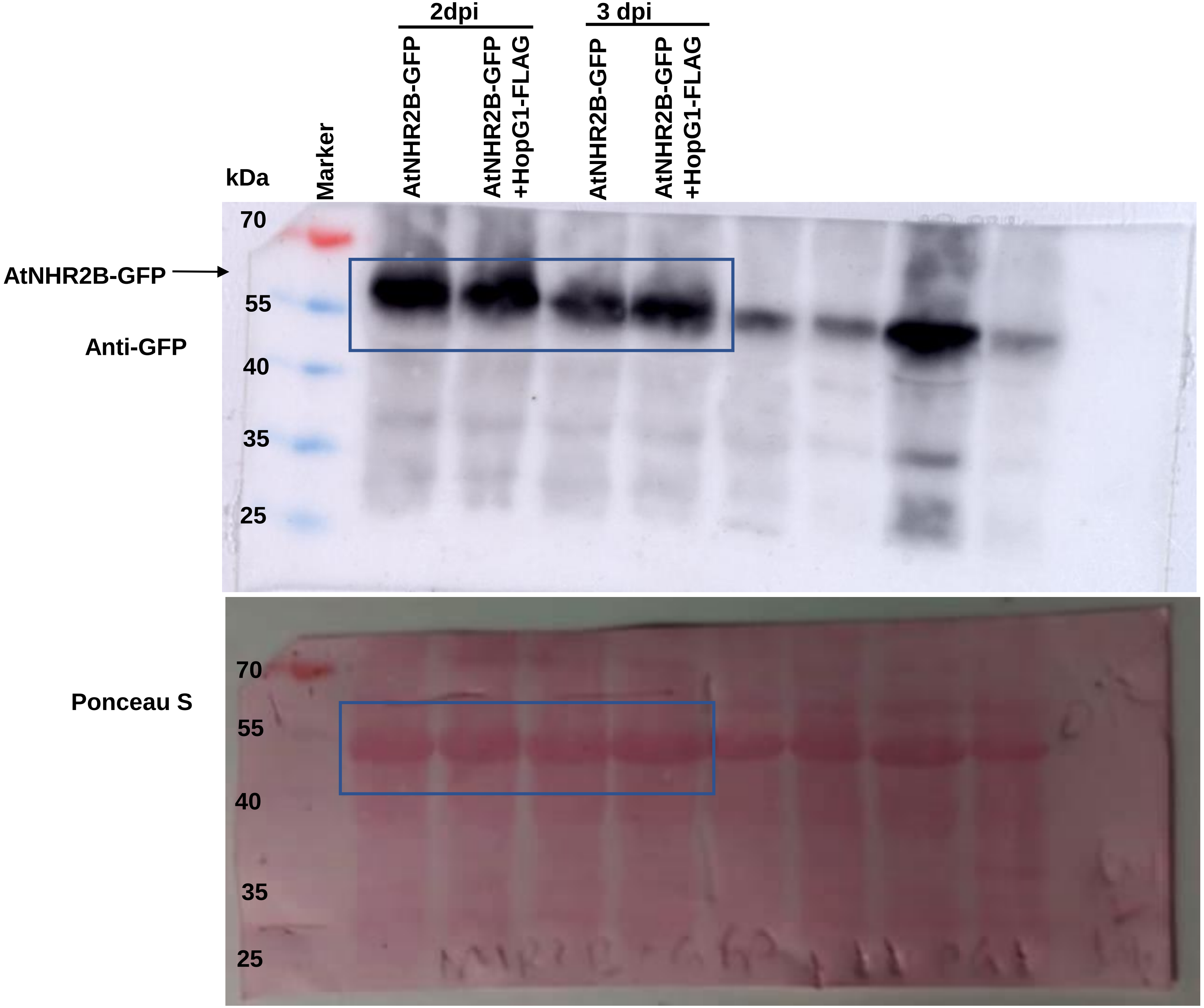


**Figure S 7.** Co-expression of *AtNHR2B-GFP* with *HopG1-FLAG* do not alter the AtNHR2B-GFP protein abundance. *AtNHR2B-GFP* was transiently expressed in *N. benthamiana* alone or with *AtNHR2B-FLAG.* At 24 hpi, 20μM DEX was sprayed to induce the expression of *HopG1-FLAG*. Infiltrated leaves were collected 72 hpi for protein extraction and Western Blot analysis using anti-GFP antibodies. Ponceau S staining showed equal protein loading. Membrane was cut before incubation with antibodies.

Table S1. Statistical significance for data presented in Figure 5c

| Time (min) | Genotype | Mean | Grouping |
|---|---|---|---|
| 1 | Col-0 | 2.6886 | B |
| | AtNHR2B-GFP | 2.2664 | B |
| | HopG1-FLAG | 3.199 | A |
| | AtNHR2B-GFP/HopG1-FLAG | 2.715 | AB |
| 2 | Col-0 | 2.275 | C |
| | AtNHR2B-GFP | 4.127 | A |
| | HopG1-FLAG | 3.919 | AB |
| | AtNHR2B-GFP/HopG1-FLAG | 3.314 | B |
| 3 | Col-0 | 2.0754 | B |
| | AtNHR2B-GFP | 2.5841 | B |
| | HopG1-FLAG | 3.522 | A |
| | AtNHR2B-GFP/HopG1-FLAG | 2.838 | AB |
| 4 | Col-0 | 2.6584 | B |
| | AtNHR2B-GFP | 2.8505 | B |
| | HopG1-FLAG | 3.522 | A |
| | AtNHR2B-GFP/HopG1-FLAG | 2.9 | B |
| 5 | Col-0 | 2.0397 | C |
| | AtNHR2B-GFP | 2.6333 | B |
| | HopG1-FLAG | 4.543 | A |
| | AtNHR2B-GFP/HopG1-FLAG | 2.408 | BC |
| 6 | Col-0 | 1.9554 | C |
| | AtNHR2B-GFP | 2.9105 | B |
| | HopG1-FLAG | 4.679 | A |
| | AtNHR2B-GFP/HopG1-FLAG | 2.239 | C |
| 7 | Col-0 | 1.988 | C |
| | AtNHR2B-GFP | 4.189 | B |
| | HopG1-FLAG | 5.7 | A |
| | AtNHR2B-GFP/HopG1-FLAG | 3.646 | B |
| 8 | Col-0 | 2.0222 | C |
| | AtNHR2B-GFP | 2.8214 | B |
| | HopG1-FLAG | 4.415 | A |
| | AtNHR2B-GFP/HopG1-FLAG | 2.836 | B |
| 9 | Col-0 | 2.275 | B |
| | AtNHR2B-GFP | 2.765 | B |
| | HopG1-FLAG | 5.314 | A |
| | AtNHR2B-GFP/HopG1-FLAG | 2.867 | B |
| 10 | Col-0 | 2.0222 | C |
| | AtNHR2B-GFP | 2.6587 | B |
| | HopG1-FLAG | 3.968 | A |
| | AtNHR2B-GFP/HopG1-FLAG | 2.527 | BC |

Comparisons among means for each time point was done using ANOVA and Tukey’s multiple comparison test at 95% confidence level. Means that do not share a letter are significantly different.

**Table S2. Bacterial strains and plasmids used in this study**

| Bacterial strains or Plasmids | Description | Source |
|---|---|---|
| *Pseudomonas syringae* pv. tabaci | Wild type, $Rif^R$ | American Type Culture Collection |
| *Pseudomonas syringae* pv. tabaci (*GFP-TIR*) | *Pseudomonas syringae* pv. tabaco containing plasmid encoding *GFP-TIR,* $Gen^R$ | This work |
| *Pseudomonas syringae* pv. tomato DC3000 | Wild type, $Rif^R$ | (Cuppels, 1986) |
| *Pseudomonas syringae* pv. tomato DC3000 Δ *hopG1* | *hopG1* deletion mutant | (Shimono et al., 2016) |
| *Agrobacterium tumefaciens* GV2260 | Non-oncogenic strain | (Deblaere et al., 1985) |
| *pMDC107*:*AtNHR2B* | AtNHR2B fused in-frame to GFP | (Singh et al., 2018) |
| *pDEST32* | Yeast two-hybrid bait vector containing *GAL4* DNA binding domain (DBD) coding sequence | Life technologies |
| *pDEST32::HopG1* | *HopG1* fused to the *GAL4* DBD | This work |
| *pDEST22* | Yeast two-hybrid prey vector containing *GAL4* Activation Domain (AD) coding sequence | Life technologies |
| pDEST22*::AtNHR2B* | *AtNHR2B* fused to the *GAL4* AD | This work |
| *pTA7001::HopG1-FLAG* | *HopG1::3X FLAG,* dexamethasone inducible | J.M. Elmore, G. Coaker |
| *pMD1::HopG1-HA* | *HopG1::HA,* 35S promoter | J.M. Elmore, G. Coaker |
| pSITEnEYP-N1::*AtNHR2B* | N-terminal in-frame fusion of *AtNHR2B* to the n-terminal half of EYFP, $Sp^R$ | This work |
| pSITEnEYFP-N1::*AtNHR2B* $_{\Delta 1\text{-}195}$ | *AtNHR2B* truncated in the first N-terminal 195 amino acids and fused to the n-terminal half of EYFP | This work |
| pSITEcEYFP-C1::*HopG1* | C-terminal in-frame fusion of *HopG1* to the c-terminal half of EYFP, $Sp^R$ | This work |

## References


Cuppels, D.A. 1986. Generation and Characterization of Tn5 Insertion Mutations in Pseudomonas syringae pv. tomato. Appl Environ Microbiol 51:323-327.

Deblaere, R., Bytebier, B., De Greve, H., Deboeck, F., Schell, J., Van Montagu, M., and Leemans, J. 1985. Efficient octopine Ti plasmid-derived vectors for Agrobacterium-mediated gene transfer to plants. Nucleic Acids Res 13:4777-4788.

Shimono, M., Lu, Y.J., Porter, K., Kvitko, B.H., Henty-Ridilla, J., Creason, A., He, S.Y., Chang, J.H., Staiger, C.J., and Day, B. 2016. The Pseudomonas syringae Type III Effector HopG1 Induces Actin Remodeling to Promote Symptom Development and Susceptibility during Infection. Plant Physiol 171:2239-2255.

Singh, R., Lee, S., Ortega, L., Ramu, V.S., Senthil-Kumar, M., Blancaflor, E.B., Rojas, C.M., and Mysore, K.S. 2018. Two Chloroplast-Localized Proteins: AtNHR2A and AtNHR2B, Contribute to Callose Deposition During Nonhost Disease Resistance in Arabidopsis. Mol Plant Microbe Interact 31:1280-1290.
